# Effects of localized and general fatigue on postural adjustments coupling during predictable external perturbations

**DOI:** 10.1007/s00421-025-05760-y

**Published:** 2025-04-05

**Authors:** Mauro Nardon, Francesco Piscitelli, Cristiano Alessandro, Enrico Tam, Matteo Bertucco

**Affiliations:** 1 Department of Neurosciences, Biomedicine and Movement Sciences, University of Verona, Via Felice Casorati 43, 37131 Verona, Italy; 2 Present Address: School of Medicine and Surgery/Sport and Exercise Medicine, University of Milano-Bicocca, Milan, Italy

**Keywords:** Postural control, Anticipatory postural adjustments, Compensatory postural adjustments, Neuromuscular fatigue, EMG

## Abstract

**Purpose:**

The central nervous system (CNS) coordinates anticipatory (APA) and compensatory postural adjustments (CPA) to face both self-induced and external perturbations. Neuromuscular fatigue (NMF), whether localized or general, impairs the CNS's ability to maintain postural stability, but the differential effects of these fatigue types on the coupling between APA and CPA remain unclear. This study aimed to investigate how localized and general NMF influence the neuromuscular control of postural adjustments during predictable external perturbations.

**Methods:**

Fourteen participants were exposed to two exercise protocols: intermittent isometric exercise to induce localized NMF and prolonged upper body exercise at high cardiometabolic effort to induce general NMF. Exercise intensity was monitored by measuring cardiometabolic parameters during exercise and recovery. Postural adjustments were assessed before and after NMF (recovery period) using electromyography and kinematic analyses while participants were exposed to predictable perturbations.

**Results:**

Localized NMF led to decreased muscle activation and co-activation across both fatigued and non-fatigued muscles during APA, with persistent kinematic changes in lower limb joints. In contrast, general NMF induced short-lived increases in EMG activity and co-activation, reflecting a strategic CNS adaptation to maintain stability.

**Conclusions:**

The results suggest that localized NMF induces a more extensive and enduring impact on postural control mechanisms, likely due to altered proprioceptive feedback, whereas general NMF effects are more transient, aligning with the rapid recovery of cardiometabolic parameters. These findings highlight the CNS's role in differentially adapting postural strategies depending on the type of fatigue, with implications for understanding how fatigue impacts stability in dynamic environments.

**Graphical abstract:**

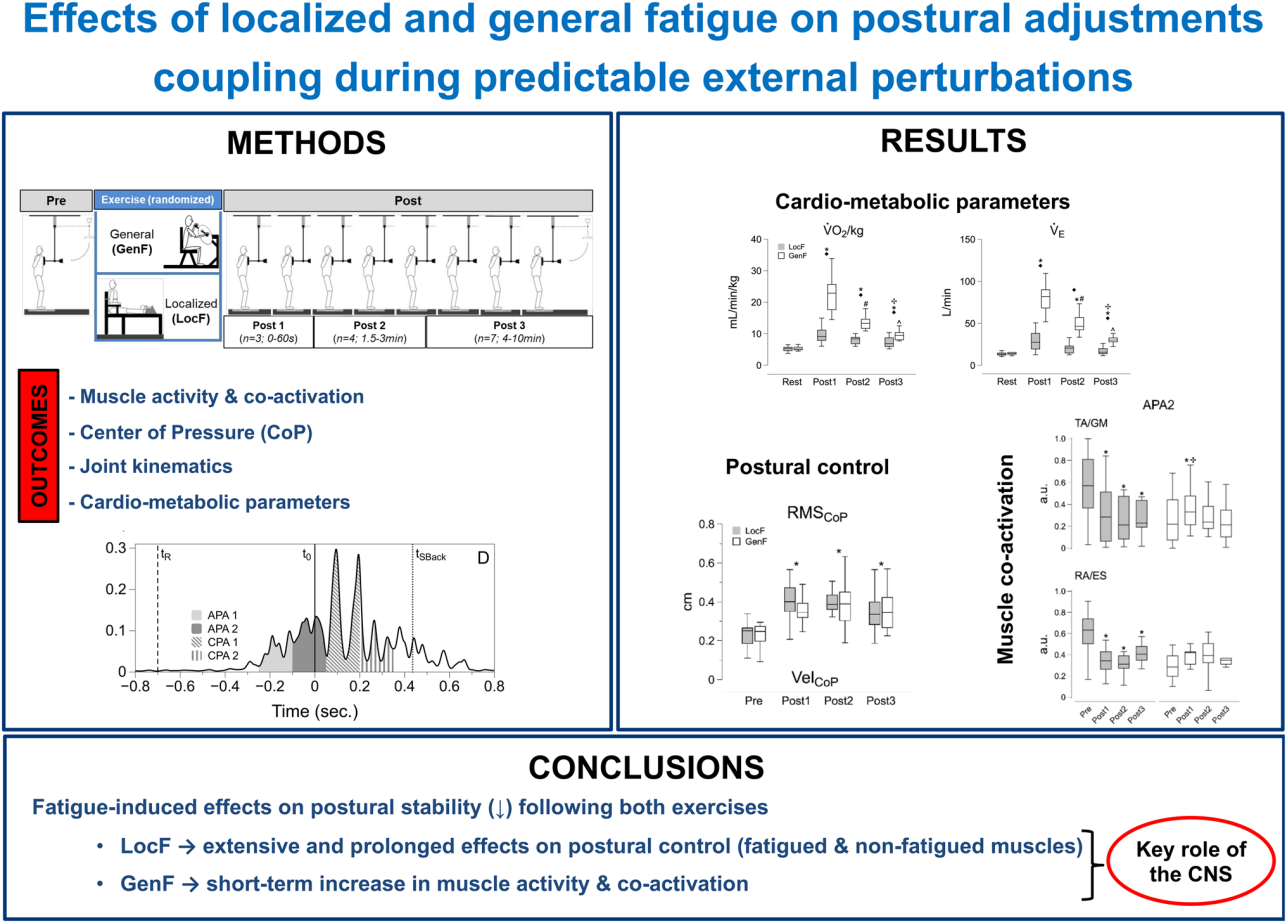

**Supplementary Information:**

The online version contains supplementary material available at 10.1007/s00421-025-05760-y.

## Introduction

Upright standing posture can be viewed as a multi-segmented inverted pendulum equipped with multiple muscles, along with their inherent viscoelastic properties. Maintaining vertical posture in the field of gravity is therefore a challenging task that requires active control, especially when a standing person performs rapid motor actions or interacts with the environment (Ivanenko and Gurfinkel 2018). There are several strategies that the central nervous system (CNS) implements to maintain posture during self-induced perturbations (such as voluntary rapid movements) or when counteracting external perturbing forces. If these perturbations are predictable, anticipatory activity is seen in postural muscles—addressed as anticipatory postural adjustments (APA), occurring up to 250 ms prior to the movement or perturbation. The function of APAs is to generate forces and torques that counteract the direct mechanical effects of the foreseeable disturbance (Belen’kiĭ et al. 1967; Massion 1992). By their nature, APA are based on predictive feedforward control and therefore there will always be residual disturbances affecting the body. These residual disturbances lead to compensatory postural adjustments (CPA) after the onset of movement or perturbation (Santos et al. 2010a, b; Krishnan et al. 2011; Chen et al. 2015). It is important to notice that APA and CPA are interconnected: when APA are not applied, CPA are the mechanism used by the CNS for restoring balance; on the other hand, when APAs are strongly involved, CPAs are less present (Santos et al. 2010a, b).

Many task contexts and internal physiological factors affect the generation of APA, such as magnitude and direction of perturbation (Aruin and Latash 1995), movement accuracy demands (Bertucco and Cesari 2010; Bertucco et al. 2013), motor actions under uncertainty (Piscitelli et al. 2017), body instability (Aruin et al. 1998; Pascucci et al. 2023), fear of falling (Adkin et al. 2002), mood state (Kitaoka et al. 2004), hypoxia (Šarabon et al. 2018).

Neuromuscular fatigue (NMF) is a transient physiological phenomenon, defined as the exercise-induced reduction in the ability of the muscle to produce force or power, which contributes to deteriorating the effectiveness of sensory and motor output (Enoka and Duchateau 2008). As a consequence, it has a detrimental effect on the sensorimotor control of movement, and therefore on the ability to maintain postural stability (Paillard 2012; Monjo et al. 2015). In the fields of exercise physiology, exercise-induced NMF can be distinguished into general and local. General fatigue involves multiple joints and muscle groups that vigorously solicits high cardiometabolic effort. On the other hand, local fatigue often involves a single joint and a limited number of muscles that strongly stimulate the neuromuscular system (Paillard 2012).

While the effects of fatigue on postural adjustments have been studied extensively, only a recent study has compared how the CNS reorganizes postural adjustments in response to local or general fatigue during a self-initiated rapid arm-raising movement (Lyu et al. 2021). Local neuromuscular fatigue was induced by sub-maximal intermittent isometric knee extensions, while general fatigue was implemented by rowing on an ergometer at a constant speed for 20 min, hence targeting mainly upper limb and trunk muscles. The results showed that APA coactivations in the trunk and thigh muscles were greater after local fatigue exercise than after general fatigue exercise, suggesting a general compensation by the CNS in response to the neuromuscular deficits in the locally fatigued muscle. The greater CPA coactivation of trunk and thigh muscles after both fatiguing exercises suggested the directional nature of muscle activation even under fatiguing conditions (Lyu et al. 2021). However, only trunk and proximal leg muscles were recorded in this study, so it remains to be clarified whether differing NMF regimes lead to different neuromuscular synergistic reorganization of muscle activations at the lower extremity joints during anticipatory and compensatory postural strategy phases. Indeed, a previous study has demonstrated that the biomechanical constraints and threat conditions influence the coupling between APA and CPA by differently controlling the coactivation of agonist–antagonist muscles at distal and proximal joints of lower extremities (Cesari et al. 2022).

Furthermore, it has been shown that the changes in APA onset latencies persisted beyond the restoration of force production after ankle muscles were locally fatigued during externally initiated perturbations (Kennedy et al. 2012). This suggests a centrally mediating protective response, as opposed to a peripherally-driven limitation in performance. Moreover, despite central and peripheral alterations in physiological processes due to NMF having been extensively studied (Carroll et al. 2017), none of the previous studies monitored cardio-metabolic parameters during the recovery from NMF, possibly linking alterations in neuromuscular strategies and exercise-induced cardio-metabolic homeostasis perturbation.

Therefore, the primary aim of the present study was to investigate the effect of different fatiguing exercise protocols (general and local fatigue) on the reorganization of muscle activity at the lower extremity joints during the ﻿anticipatory and compensatory phases of postural adjustments. Participants were exposed to a predictable external perturbation before and after inducing neuromuscular fatigue. Since the mechanical characteristics of the external perturbation will not change, any detectable alteration to the APA and CPA would solely underlie the effect of fatigue on the feedforward and feedback postural control mechanisms (Santos et al. 2010a; Chen et al. 2017; Cesari et al. 2022). We hypothesized that localized NMF will affect primarily proximal postural muscles, leading to an increase in muscle activity and coactivation level during APA. Whereas we expect that general fatigue will increase muscle activity and co-contraction in both proximal and distal muscles. These differences were expected to persist in the CPA, suggesting a fatigue-specific reorganization of neuromuscular control in response to predictable external perturbations.

As a second aim, we examined the differences in the adaptation of anticipatory and compensatory postural mechanisms during the recovery period after general (exercise with high cardiometabolic effort) and localized NMF, simultaneously monitoring the physiological recovery of cardio-metabolic parameters. Thus, we hypothesized that the time-dependent recovery of general and localized NMF would lead to distinct adaptive postural strategies following fatiguing exercise. Specifically, the increased metabolic response during general NMF was expected to influence postural control, likely mirroring the rapid recovery of cardio-metabolic parameters. In contrast, we anticipate that localized NMF will have a more extensive and enduring impact on postural control mechanisms.

## Methods

### Participants

Fourteen healthy male subjects without a history of cardiovascular disease and musculoskeletal injuries to the limbs were recruited for this study (age: 25.3 ± 4.2 years; height: 1.78 ± 0.06 m; weight: 77.1 ± 6.8 kg). The recruitment of only male participants was based on methodological and physiological considerations. The postural perturbation involved a weighted pendulum impacting the chest with a force of approximately 140 N. Due to anatomical differences, including soft tissue composition, female participants were excluded to avoid variability in responses. Participants had normal or corrected to normal vision. The study protocol conformed to the principles of the Declaration of Helsinki and was approved by the local Ethical Committee (Prot. N°13/2019). Participants provided written informed consent before taking part in the study.

### Study design

Participants were exposed to two different exercise protocols in a crossover design: intermittent isometric exercise to induce localized NMF (LocF) and prolonged exercise with upper extremities at high cardiometabolic effort, inducing general NMF (GenF). Both exercise protocols are detailed later in the Fatiguing Exercise Protocols section. Participants were asked to come to the laboratory for assessments at two different visits with at least 72 h interval between them. During each visit, participants performed repetitions of the same postural tasks before (pre) and after (post) either LocF or GenF. The order of the exercise protocol was randomized across subjects. Participants were granted 10 trials at the beginning of each session to familiarize with the task and then performed 12 trials prior to the fatiguing exercise (Pre). Participants performed 14 trials during the following 10 min of recovery (one trial every 30 s for the first 3 min, 1 every 60 s for the following minutes). The time intervals between trials were based on pilot tests, where few participants experienced a light-headed state after switching from the GenF protocol to a standing static position.

### Postural task

**Fig. 1:**
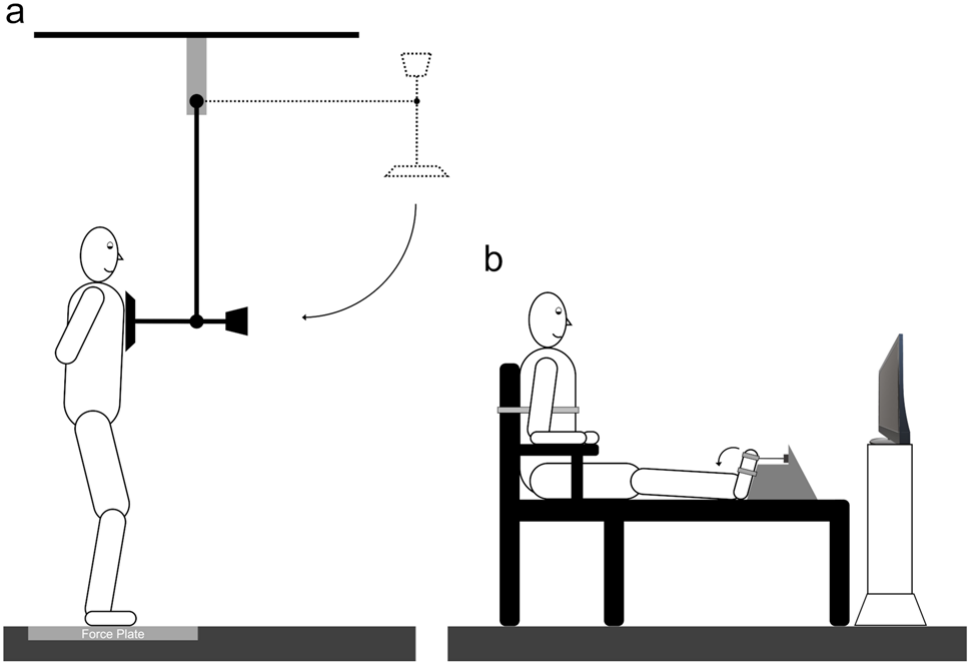
**a** Schematic diagram of the experimental set-up. Participants were asked to stand upright barefoot on a force plate in front of a pendulum with their hands crossed behind their back. They were instructed to receive a series of perturbations coming from the front and to maintain their balance after the perturbation. **b** Set-up for the localized neuromuscular fatigue exercise (LocF)

Participants were asked to stand upright barefoot in front of a pendulum with knees slightly bent and their hands crossed behind their back (Fig. 1a). They were instructed to receive a series of pendulum perturbations directed frontally to their chest, and to maintain their balance after each perturbation for at least 5 s. A weighted pad (total mass: 0.75 kg), connected to the fulcrum by a 1-m aluminum bar was kept horizontal (90 ° respect to the impact position) and released by an experimenter randomly within 5 s after an auditory cue. Fulcrum’s height was adjusted to have the pad impacting at the participant’s sternum level and with the pendulum perpendicular to the ground. The magnitude of the pendulum impact on the participants’ chest was about 140 N (Fig. 1a). A tri-axial accelerometer (CZL635, Robot Italy srl, Rome, Italy, sampling rate: 2000 Hz) was secured to the pendulum to detect its motion. Participant’s feet position was marked on the ground and kept constant across trials. The experimental procedure was specifically designed to avoid the involvement of the upper limbs in the postural task.

### Fatiguing exercise protocols

When performing the LocF exercise protocol, participants were seated comfortably on a chair with their backs rested, the knees fully extended, ankles in plantar flexion at 10° and the feet fastened together with nylon straps to a custom-made setup (Fig. 1b). The custom-made setup was connected through an inextensible cable to a load cell (System Pese, Milan, Italy; linear response: 1500N, sampling frequency: 1000 Hz). Participants initially performed 3 maximal voluntary contractions (MVC) in ankle dorsiflexion, interleaved by 2 min of rest. Then they performed a submaximal intermittent isometric exercise with the dorsiflexors muscle groups (60 ± 5% MVC; 45 s, duty cycle: 0.67; contraction/relaxation: 30 s/15 s). The sounds of a timer set the pace of the exercise and visual feedback of force production relative to the target force was provided on a screen in front of participants. Participants were constantly motivated throughout the exercise. Task failure was determined when participants did not reach and hold for more than 1 s the target force for two consecutive cycles. The ankle dorsiflexors were chosen as the target muscles for the LocF exercise because of their primary contribution to maintaining balance when receiving the pendulum perturbation (Santos et al. 2010a; Kaewmanee et al. 2020; Cesari et al. 2022).

The GenF exercise consisted of a step-incremental test on an electromagnetically braked arm ergometer (ergoselect 400, ergoline GmbH, Bitz, Germany). The ergometer was connected to and operated by a PC running the metabolimeter software (Omnia™, Cosmed, Rome, Italy) allowing to impose workloads according to predefined protocols. Participants were at rest for 2 min (rest), warmed up at 50 Watts (W) for 3 min, then the workload increased by 10 W/min until participants could no longer sustain the exercise. Participants were constantly motivated and reminded to maintain the cadence within 70–80 revolutions per minute throughout the exercise. Task failure was determined when participants’ cadence fell below 55 revolutions per minute despite verbal encouragement.

Distance between both exercise protocols’ setup and the postural task setup was minimized to allow participants to perform the subsequent trials post-exercise (Post) immediately after the termination of the protocol (≤ 5 s).

### Kinematics and posturography

Kinematic data were collected at 250 Hz using an 8-camera motion capture system (MX 13, VICON, Oxfordshire, UK). Sixteen reflective markers (14 mm in diameter) were placed on the following anatomical landmarks of both sides of the body: heel (calcaneus), tip toe (distal phalanx), ankle joint (lateral malleolus), knee joint (lateral epicondyle of femur), hip joint (great trochanter), shoulder (acromion) and two additional markers on the mid-point between ankle-knee and knee-hip joints, respectively to improve the reconstruction of the 2-D model. Before starting the session, a static trial was performed to reconstruct the model and label each marker for automatic software detection during the trials. The center of pressure (CoP) displacement while standing in front of the pendulum was recorded by means of a force platform (model OR-5, AMTI, USA: 90 × 90 cm, sample rate: 2000 Hz).

### Surface EMG

Surface EMG signals were recorded from 8 muscles on participants’ dominant side using a wireless system (Zerowire, Aurion, Italy). After proper shaving and cleansing of the skin with alcohol swabs, Ag/AgCl electrodes (PG10C; Fiab, Vicchio, Italy) were attached to the skin with a 20-mm interelectrode distance. Electrodes were positioned over six muscles belly on the dominant side of the body, following recommendations (Hermens et al. 2000): rectus femoris (RF), biceps femoris (BF), tibialis anterior (TA), gastrocnemius medialis (GM), rectus abdominis (RA) and erector spinae (ES). Electrode placement was confirmed by asking the participants to perform a set of isometric contractions and related free movements while observing the resulting EMG patterns (Kendall et al., 2005). EMG signals were sampled at 2000 Hz. Motion capture, force plate and EMG system were synchronized with a hardware device (MX Control, VICON, Oxfordshire, UK) that matched the data acquisition across systems.

### Cardio-metabolic parameters

Cardio-ventilatory parameters were collected for 2 min prior exercise (Rest), throughout the exercise phase (exercise) and for 10 min after the end of the exercise (Post-corresponding to the post trials in the postural task) using a portable breath-by-breath metabolic system (K5, Cosmed, Rome, Italy), calibrated following manufacturer’s instructions.

### Data processing

#### Cardio-metabolic data analysis

Breath-by-breath absolute values (litre/min) of oxygen consumption (${\dot{\text{V}}\text{O}}_{2}$), carbon dioxide production (${\dot{\text{V}}\text{CO}}_{2}$) and pulmonary ventilation ($\dot{V}_{{\text{E}}}$) were obtained by means of manufacture software (Omnia™, Cosmed, Rome, Italy) together with heart rate (HR) collected by telemetry Polar^®^ wireless band (beats/min). Also, respiratory rate (RR) (l/min) has been obtained. ${\dot{\text{V}}\text{O}}_{2}$ was normalized by body mass unit (${\dot{\text{V}}\text{O}}_{2}$/kg). The mechanical load (Watt) of the arm ergometer was recorded continuously by the software running the metabolimeter. Resting data (Rest) was calculated as the mean values of the last 30 s before the warm-up phase begins; ${\dot{\text{V}}\text{O}}_{2}$ max and all maximal variables were calculated as the mean of the last 30 s before the end of the exercise. All the metabolic variables were exported as spreadsheet files and averaged over 5 s to get all metabolic data aligned with the starting of the recovery phase. Each ${\dot{\text{V}}\text{O}}_{2}$ five sec data was further averaged every 15 (data discrete binning) around each impact of the pendulum. The same procedure was done for: ${\dot{\text{V}}\text{CO}}_{2}$, $\dot{V}_{{\text{E}}}$_,_ HR, and RR. Each participant's data series were then ensemble averaged at the following time points after the fatiguing protocols: Post1 (including data at time 0—i.e., immediately after the end of the exercise, 30, and 60 s), Post2 (including data at time 1.5, 2, 2.5, and 3 min), Post3 (including data at time 4, 5, 6, 7, 8, 9, and 10 min).

#### Kinematics and posturography data analysis

**Fig. 2:**
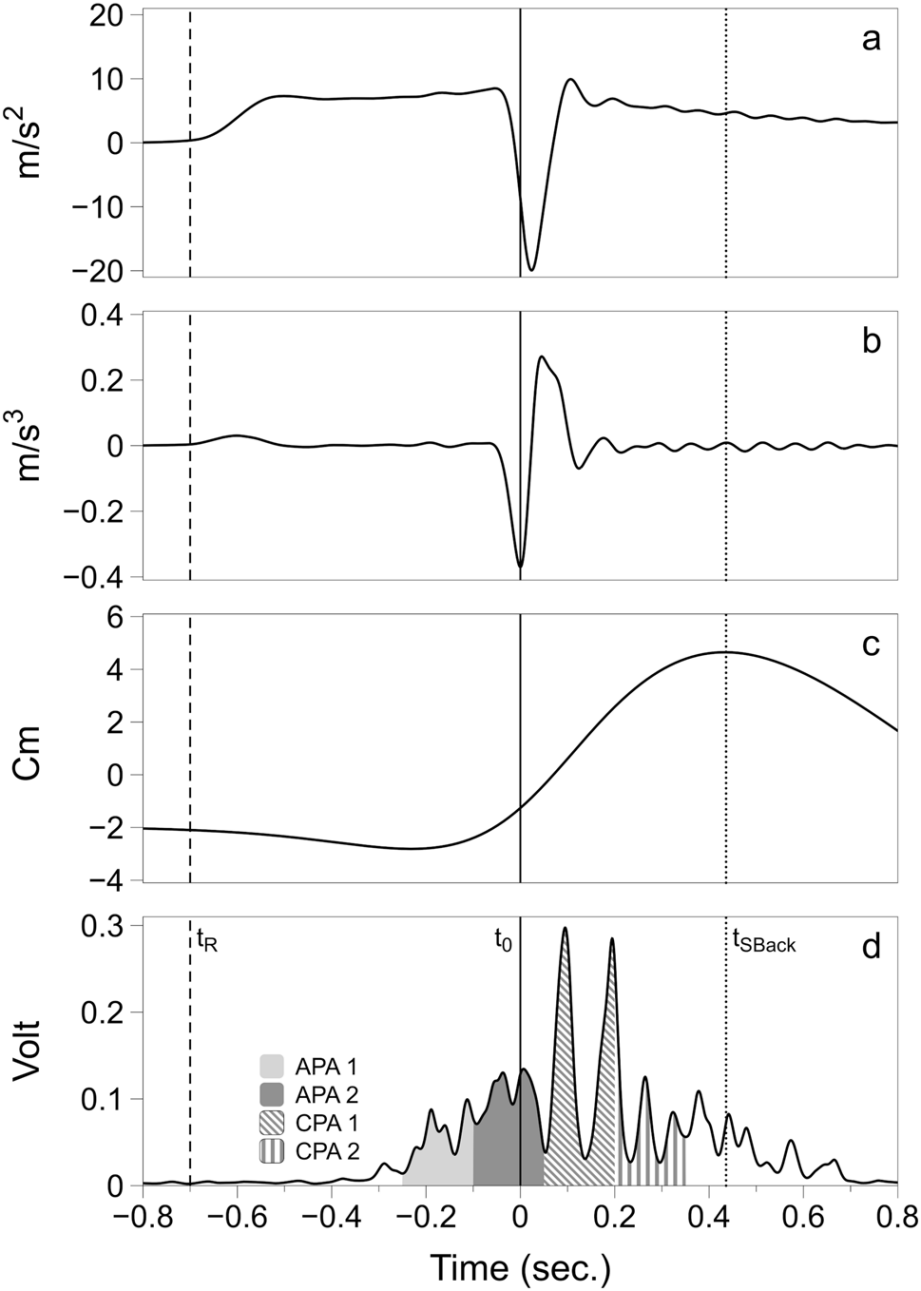
A representative trial showing: **a** the acceleration of the pendulum in antero-posterior direction; **b** the 1st derivative of the acceleration of the pendulum; **c** the displacement of the shoulder of the dominant side in antero-posterior direction; **d** filtered EMG trace of the tibialis anterior (TA) of the dominant lower limb. APA1, APA2, CPA1, CPA2 are highlighted in light gray, dark gray, oblique and vertical lines pattern respectively. The solid, dashed, and dotted lines represent the *t*_0_ (time of impact), *t*_R_ (time of release) and *t*_Sback_ (timepoint of maximum shoulder displacement), respectively

Kinematic data were reconstructed using Vicon Nexus software (version 2.12, VICON, Oxfordshire, UK), then offline processed together with all the other data using customized scripts in MATLAB software (R2021b, version 9.11.0, MathWorks, Natick, MA, USA). Load cell, force plate and kinematics data were low pass filtered (4th order Butterworth with cut-off frequencies of 10 Hz). The signal obtained from the accelerometer attached to the pendulum was used to identify task-related events: (1) the timepoint when the pendulum impacted with the participant’s chest (*t*_0_) was defined as the time of the negative peak of the first derivative of the acceleration signal in the antero-posterior direction, (2) the time of the release (*t*_R_) of the pendulum was calculated as the instant when the magnitude of the accelerometer signal in the antero-posterior direction crossed 5% of its maximal value before *t*_0_ (Cesari et al. 2022). The maximum shoulder displacement in the sagittal plane after *t*_0_ (*S*_Back_) was measured to assess the effect of the perturbation on upper body kinematics following the impact. It was calculated as the distance covered by the marker attached to the acromion of the dominant side between *t*_0_ and the instant when its tangential velocity crossed the zero value after *t*_0_ (Cesari et al. 2022). The instant in time of *S*_Back_ was also computed (*t*_SBack_) (Fig. 2).

The kinematics of relative joint angles of the dominant side in the sagittal plane were computed for the ankle (*θ*_Ankle_), knee (*θ*_Knee_) and hip (*θ*_Hip_) joints at three different time points: at pendulum release (*t*_R_), at the instant of impact (*t*_0_) and at the time when the maximum shoulder displacement happens following the impact (*t*_SBack_). The magnitude and polarity of each joint angle were described according to Perry and Burnfield (2010) (Perry and Burnfield 2010). Specifically, for the ankle joint, 90° between the shank and the foot was the boundary between plantarflexion (*θ*_Ankle_ < 0) and dorsiflexion (*θ*_Ankle_ > 0). When the knee was fully extended, it was described as 0° flexion, and when the shank moved to a posterior direction relative to the thigh, the knee joint angle was said to be in flexion (*θ*_Knee_ > 0). The *θ*_Hip_ was defined by the path of thigh displacement from the vertical with positive angles for hip flexion and negative angles for hip extension.

To assess the effect of the fatiguing protocols on the upright standing posture, we examined the CoP displacement before the impact during a time window of 2 s before *t*_R_. Specifically, we computed the root mean square (RMS_CoP_) and mean velocity (Vel_CoP_) of the CoP in both antero-posterior and medio-lateral directions (Raymakers et al. 2005; Duarte and Freitas 2010) as follows:

$$\begin{aligned} {\text{RMS}}_{{{\text{COP}}}} & = \sqrt {\left( {\sqrt {\frac{{\left( {{\text{CoPx}}_{1}^{2} + {\text{CoPx}}_{2}^{2} + \cdots + {\text{CoPx}}_{n}^{2} } \right)}}{n}} } \right)^{ 2} + \left( {\sqrt {\frac{{\left( {{\text{CoPy}}_{1}^{2} + {\text{CoPy}}_{2}^{2} + \cdots + {\text{CoPy}}_{n}^{2} } \right)}}{n}} } \right)^{2} } \\ {\text{Vel}}_{{{\text{CoP}}}} & = \frac{{\mathop \sum \nolimits_{i = 1}^{n} \frac{{\sqrt {\left( {{\text{CoPx}}_{i} - {\text{CoPx}}_{i - 1} } \right)^{2} + \left( {{\text{CoPy}}_{i} - {\text{CoPy}}_{i - 1} } \right)^{2} } }}{{t_{i} - t_{i - 1} }}}}{n} \\ \end{aligned}$$

where CoPx and CoPy are the de-meaned signals of CoP in medio-lateral and antero-posterior directions respectively, n is the number of samples. Note that for CoPx and CoPy with zero mean, RMS_CoP_ equals the planar standard deviation (i.e., considering both medio-lateral and antero-posterior directions) (Raymakers et al. 2005; Duarte and Freitas 2010).

#### EMG data analysis

EMG data were firstly detrended, band-pass filtered (5–450 Hz; 4th order Butterworth) and a notch filter (47–53 Hz, 4th order Butterworth) was applied to remove power-line noise. Resulting EMG signals were then separately processed for the determination of the timing of activation (onset) and the magnitude of the activity. Additionally, EMG median frequency of TA muscle during the fatiguing exercises was computed to confirm fatigue-induced changes in muscle spectral power frequency following the LocF protocol. To determine the onset of EMG activity bursts, signals were then high-pass filtered (20 Hz, 6th order Butterworth) to remove movement artifacts (Solnik et al. 2008, 2010). Onsets for TA (Onset_TA_), RF (Onset_RF_) and RA (Onset_RA_) were derived from the filtered EMG signal using the Teager–Kaiser onset time detection method; i.e. they were defined as the timepoint in which the EMG amplitude overcame and remained greater for at least 50 ms than the mean ± 2 SD of the baseline activity measured from − 2100 to − 2000 ms with respect to *t*_0_ (Li et al. 2007; Curuk et al. 2020). A priori analyses showed a primary contribution of these muscles when receiving the load, which was also confirmed by previous studies using the same type of perturbation (Santos et al. 2010a; Kaewmanee et al. 2020; Cesari et al. 2022).

To quantify the magnitude of EMG activity, the band- and notch-pass filtered signals were subjected to full-wave rectification, followed by low-pass filtering (20 Hz, 4th order Butterworth) (Kaewmanee et al. 2020) to obtain EMG envelopes. EMG activity of all studied muscles (ʃEMG) was calculated using a trapezoidal numerical integration (function trapz in Matlab) during the four epochs (relative to *t*_0_): (1) from − 250 to − 100 ms (anticipatory postural adjustment—window 1; APA1), (2) from − 100 to + 50 (anticipatory postural adjustment—window 2; APA2), (3) from + 50 to + 200 ms (compensatory postural adjustment—window 1; CPA1), (4) from + 200 to + 350 ms (compensatory postural adjustment—window 2; CPA2). Background EMG activity (EMGbackground) was computed from a 50 ms window at the beginning of each trial—EMG signal was visually inspected to confirm the absence of abnormal activity—and subtracted to correct each epoch integral:

$$\begin{aligned} \int APA1_{i} & = \mathop \int \limits_{ - 250}^{ - 100} EMG_{i} - 3 \int EMGbackground_{i} \\ \int APA2_{i} & = \mathop \int \limits_{ - 100}^{ + 50} EMG_{i} - 3 \int EMGbackground_{i} \\ \int CPA1_{i} & = \mathop \int \limits_{ + 50}^{ + 200} EEMG_{i} - 3 \int EMGbackground_{i} \\ \int CPA2_{i} & = \mathop \int \limits_{ + 200}^{ + 350} EMG_{i} - 3 \int EMGbackground_{i} \\ \end{aligned}$$

where *i* represents the EMG signal for each muscle.

For comparison across subjects, EMG values were normalized by the highest positive value (ʃEMGmax) within conditions, for each muscle and each subject separately (Slijper and Latash 2000, 2004). Note that after this normalization, integrated EMG values were comprised within the range from + 1 to − 1, where negative values corresponded to a decrease in the background activity with respect to the background window.

Furthermore, we computed indexes of co-activation (C-index) within agonist–antagonist muscle pairs (TA/GM, RF/BF and RA/ES) acting at a joint level within the framework of the equilibrium-point hypothesis (Feldman 1986). Indexes were computed using EMG integrals of ventral and dorsal muscles for each time-epoch (Slijper and Latash 2000, 2004). Specifically, C-Index = 0 if ∫EMG_ventral_ and ∫EMG_dorsal_ had different signs; C-Index = min {|∫EMG_ventral_|;|∫EMG_dorsal_|} if ∫EMG_ventral_ and ∫EMG_dorsal_ had the same signs (Piscitelli et al. 2017; Cesari et al. 2022; Pascucci et al. 2023; Nardon et al. 2024b).

Equally to cardio-metabolic variables, all the outcome variables considered for the kinematic, posturography and EMG analysis were averaged at the three timepoints after the fatigue exercise: Post1 (including data at time 0, 30, and 60 s), Post2 (including data at time 1.5, 2, 2.5, and 3 min), Post3 (including data at time 4, 5, 6, 7, 8, 9, and 10 min).

### Statistical analysis

Data are presented in the figures either as bar plots depicting mean ± 1 standard error of the mean (SEM) bars, or as box plots depicting the median and the 25th and 75th quartiles and the whisker showing the min and max values. Skewness and kurtosis were used to assess the normality of the data. Two-way repeated-measures ANOVAs were performed to compare the Pre and Post conditions for each fatiguing protocol. As fixed effects, we used factors Condition [2 levels: GenF and LocF] and Time [4 levels: Pre or Rest, Post1, Post2 and Post3] for RMS_CoP_, Vel_CoP_, joint kinematics and the cardiometabolic variables during recovery. Two-way repeated-measures ANOVAs were used to compare the cardiometabolic responses of the two fatiguing exercise protocols at rest (Rest) and at task failure (Max). Thus, as fixed effects, we considered Condition [2 levels: GenF and LocF] and Time [2 levels: Rest and Max (task failure)]. Since the EMGs were recorded and normalized within each of the two fatigue sessions, one-way repeated-measures ANOVAs were performed to compare the factor Time [4 levels: Pre, Post1, Post2 and Post3], within each ∫EMG epoch (∫APA1, ∫APA2, ∫CPA1, ∫CPA2) separately. Similarly, C-Index were compared across Time for each muscle pairs (TA/GM, RF/BF and RA/ES) within each fatigue session. To assess the effect of the submaximal intermittent isometric exercise on LocF, a paired-sample t-test was performed for the mean force produced (mF) between the first and last isometric sustained contraction (30 s). Similarly, EMG median frequency of the TA muscle was compared between the first and the final contraction in the fatiguing protocol, for each session separately. Pairwise comparisons with Tukey’s HSD corrections were used to explore significant effects. A Friedman non-parametric test was performed in case the normality of the data was not verified, and Durbin-Conover test was used for multiple comparisons. A significance level of *p* < 0.05 was set for all statistical tests. Statistical analysis was performed using Jamovi software (The jamovi project (2024), Version 2.3.21 https://www.jamovi.org). All figures were created using DataGraph^®^ (Version 5.4, Visual Data Tools Inc., Chapel Hill, NC, USA).

## Results

### Metabolic responses to the fatiguing protocols

The average maximum force exerted by the ankle dorsiflexors during MVC prior to the LocF fatigue exercise was 485.2 ± 81.9 N, with an average time to task failure of 592 ± 170 s. The paired t-test revealed a significant decrease in mF (*t*_(13)_ = 10.7, *p* < 0.001) between the first ($\overline{x}$ = 284.7 ± 47.6 N) and last ($\overline{x}$: 192.9 ± 23.5 N) isometric sustained contraction, resulting in a mean drop in force of 32 ± 0.07%. As concerns the step-incremental test to induce GenF, the maximal power output reached by the participants on the ergometer was on average 118.7 ± 23.8 W. The time to exhaustion was on average 574 ± 142 s including the 3 min of warm-up. Power spectral median frequency of TA muscle decreased significantly following LocF (1st cycle: 79.1 ± 15.4 Hz—last cycle: 66.3 ± 9.8 Hz, *t*_(13)_ = 3.18, *p* = 0.007, *d* = 0.85 [CI: 0.22–1.45]), while it did not change following GenF (1st step: 99.3 ± 35.0 Hz—last step: 95.0 ± 26.8 Hz, *t*_(13)_ = 0.59, *p* = 0.566, *d* = 0.16 [CI: − 0.37–0.68]).

The two-way repeated measures ANOVAs showed significant effects of the main factors, Condition and Time, as well as of the interaction term on ${\dot{\text{V}}\text{O}}_{2}$/kg, ${\dot{\text{V}}\text{CO}}_{2}$, $\dot{V}_{{\text{E}}}$ and HR during exercise (Table 1), showing that the GenF protocol induced significantly higher responses for all the metabolic parameters compared to LocF (S1 in Supplementary data).

**Table 1:** Two-way repeated measures ANOVA results for cardiometabolic parameters during exercise (Rest vs. Max—exhaustion)

Exercise							
Variable	Condition	Rest	Max		Main effects		
					*F*	*p*	*η*^2^
${\dot{\text{V}}\text{O}}_{2}$/kg (mL/min/kg)	LocF	5.18 ± 0.87	12.2 ± 5.07◆*	Condition	*F*_1,13_ = 126.9	*p* < 0.001	*η*^2^ = 0.17
	GenF	5.27 ± 0.83	30.4 ± 6.10◆*	Time	*F*_1,13_ = 178.5	*p* < 0.001	*η*^2^ = 0.53
				Condition*Time	*F*_1,13_ = 142.2	*p* < 0.001	*η*^2^ = 0.17
${\dot{\text{V}}\text{CO}}_{2}$(mL/min)	LocF	361 ± 69.7	1086 ± 504◆*	Condition	*F*_1,13_ = 122.1	*p* < 0.001	*η*^2^ = 0.17
	GenF	377 ± 59.7	2813 ± 414◆*	Time	*F*_1,13_ = 273.9	*p* < 0.001	*η*^2^ = 0.57
				Condition*Time	*F*_1,13_ = 130.8	*p* < 0.001	*η*^2^ = 0.17
$\dot{V}_{{\text{E}}}$(L/min)	LocF	13.6 ± 2.12	52.8 ± 31.0◆*	Condition	*F*_1,13_ = 67.5	*p* < 0.001	*η*^2^ = 0.11
	GenF	15.2 ± 3.36	110 ± 27.0◆*	Time	*F*_1,13_ = 90.1	*p* < 0.001	*η*^2^ = 0.58
				Condition*Time	*F*_1,13_ = 65.8	*p* < 0.001	*η*^2^ = 0.10
HR (beats/min)	LocF	77.9 ± 13.0	114 ± 19.2◆*	Condition	*F*_1,13_ = 96.7	*p* < 0.001	*η*^2^ = 0.12
	GenF	77.2 ± 8.59	172 ± 10.4◆*	Time	*F*_1,13_ = 463.0	*p* < 0.001	*η*^2^ = 0.64
				Condition*Time	*F*_1,13_ = 266.9	*p* < 0.001	*η*^2^ = 0.13

LocF: localized fatigue; GenF: general fatigue; *η*^2^ eta squared effect size. ◆ Significant effects within Condition factor (GenF vs. LocF)

*Significant effects within Time factor (Rest vs. Max). Significance level set at *p* < 0.05

The metabolic responses were compared before (Rest) and after the fatiguing exercise (recovery), separated into three timepoints (Post1, Post2 and Post3, see Methods for details) while participants received the pendulum perturbations. The ANOVAs indicated significant effects of Condition and Time, as well as of their interaction on all the considered metabolic responses (Table 2): ${\dot{\text{V}}\text{O}}_{2}$/kg, $\dot{V}_{{\text{E}}}$, RR, and HR. The metabolic responses observed following the GenF exercise remained higher than those observed following LocF, throughout the time periods. All the responses to GenFgradually decreased during recovery. LocF resulted in a less pronounced decrease in the cardio-metabolic responses during the recovery phases, with full recovery at the final phase for ${\dot{\text{V}}\text{CO}}_{2}$, and RR (Table 2 and S2 in Supplementary data).

**Table 2:** Two-way repeated measures ANOVA results for cardiometabolic parameters at rest and during recovery (Post1, Post2 and Post3)

Recovery									
Variable	Condition	Rest	Post1	Post2	Post3	Main effects			
							*F*	*p*	*η*^2^
${\dot{\text{V}}\text{O}}_{2}$/kg (mL/min/kg)	LocF	5.18 ± 0.87	10.05 ± 2.97◆*	8.32 ± 1.8◆*	7.27 ± 1.66◆*✣	Condition	*F*_1,13_ = 126.9	*p* < 0.001	*η*^2^ = 0.17
	GenF	5.27 ± 0.83	22.49 ± 5.15◆*	13.7 ± 2.21◆*#	9.61 ± 1.64◆*✣^	Time	*F*_1.5,19.1_ = 178.5	*p* < 0.001	*η*^2^ = 0.53
						Condition*Time	*F*_1.5,19.8_ = 142.2	*p* < 0.001	*η*^2^ = 0.17
${\dot{\text{V}}\text{CO}}_{2}$(mL/min)	LocF	361.34 ± 69.69	720.51 ± 208.77◆*	498.57 ± 112.4◆#	419.43 ± 83.24◆✣	Condition	*F*_1,13_ = 122.1	*p* < 0.001	*η*^2^ = 0.17
	GenF	377.04 ± 59.7	2072.95 ± 363.96◆*	1234.42 ± 171.42◆*#	729.93 ± 76.8◆*✣^	Time	*F*_1.5,19.5_ = 273.9	*p* < 0.001	*η*^2^ = 0.57
						Condition*Time	*F*_1.3,16.6_ = 130.8	*p* < 0.001	*η*^2^ = 0.17
$\dot{V}_{{\text{E}}}$(L/min)	LocF	13.59 ± 2.12	31.11 ± 15.45◆*	20.29 ± 6.07◆	16.65 ± 3.95◆*✣	Condition	*F*_1,13_ = 67.5	*p* < 0.001	*η*^2^ = 0.11
	GenF	15.18 ± 3.36	79.58 ± 17.81◆*	48.83 ± 11.35◆*#	31.01 ± 5.5◆*✣^	Time	*F*_1.3,17.4_ = 90.1	*p* < 0.001	*η*^2^ = 0.58
						Condition*Time	*F*_1.31,17.1_ = 65.8	*p* < 0.001	*η*^2^ = 0.10
RR (breaths/min)	LocF	20.72 ± 3.97	28.51 ± 12.09◆	23.76 ± 8.77◆	21.14 ± 6.83◆	Condition	*F*_1,13_ = 67.5	*p* < 0.001	*η*^2^ = 0.11
	GenF	18.65 ± 4.13	38.55 ± 8.64◆*	31.83 ± 7.39◆*#	26.86 ± 5.25◆*✣^	Time	*F*_1.4,18.3_ = 90.1	*p* < 0.001	*η*^2^ = 0.58
						Condition*Time	*F*_1.6,21.0_ = 65.8	*p* < 0.001	*η*^2^ = 0.10
HR (beats/min)	LocF	77.92 ± 13.05	104.87 ± 19.59◆*	92.49 ± 16.31◆*#	86.5 ± 13.38◆*✣^	Condition	*F*_1,13_ = 96.7	*p* < 0.001	*η*^2^ = 0.12
	GenF	77.22 ± 8.59	156.49 ± 12.64◆*	128.2 ± 13.27◆*#	109.46 ± 11.88◆*✣^	Time	*F*_1.8,23.8_ = 463.0	*p* < 0.001	*η*^2^ = 0.64
						Condition*Time	*F*_2,26.4_ = 266.9	*p* < 0.001	*η*^2^ = 0.13

LocF: localized fatigue; GenF: general fatigue; *η*^2^ eta squared effect size

◆Significant effects between Condition factor (GenF vs. LocF)

*Significant effects between Rest and Post1, Post2 or Post3;

^**#**^Significant effect between Post1 and Post2;

**✣**Significant effect between Post1 and Post3

**^**Significant effect between Post2 and Post3. Significance level was set at *p* < 0.05

### CoP displacement during upright steady-state posture

**Fig. 3:**
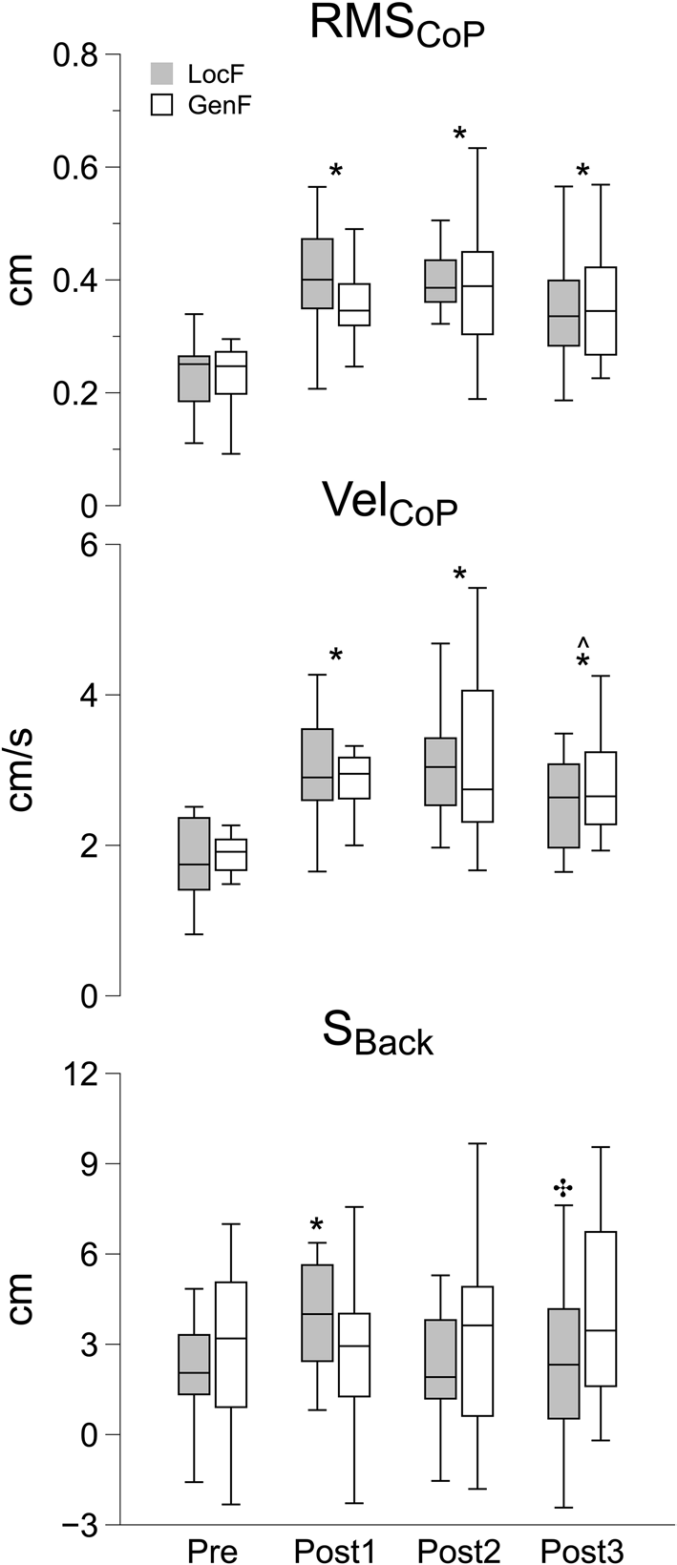
RMS_CoP_ (on the top), Vel_CoP_ (in the middle) and *S*_Back_ (at the bottom) before (Pre) and after the fatigue exercise separated by the three Post phases (Post 1, Post2 and Post3). RMS_CoP_ and Vel_CoP_ were computed over a 2-s time window before the release of the pendulum (*t*_R_). *S*_Back_ corresponds to the maximum displacement of the shoulder following the impact of the pendulum (see “Methods”–“Data processing” sections). White and grey box indicate the GenF and LocF exercise conditions respectively. * Significant effects between Pre and Post1, Post2 or Post3; ^ significant effect between Post2 and Post3; ✣ significant effect between Post1 and Post3. Significance level was set at *p* < 0.05

Root mean square (RMS_CoP_) and mean velocity (Vel_CoP_) of the CoP displacement before the release of the pendulum (*t*_R_) were calculated to assess the effect of fatigue protocols on upright standing posture. The two-way ANOVAs found significant effects of main factor Time on both RMS_CoP_ (*F*_3,38_ = 16.0, *p* < 0.001, *η*^2^ = 0.25) and Vel_CoP_ (*F*_1.98,25.8_ = 17.8, *p* < 0.001, *η*^2^ = 0.27). No significant effects were found for the factor Condition and for the interaction term. The pairwise comparisons showed significantly higher RMS_CoP_ and Vel_CoP_ during the three Post phases compared to the Pre condition (*p* < 0.01) and between Post2 and Post3 for Vel_CoP_ (*p* < 0.01), indicating that both fatigue exercises induced similar sustained decrease of postural stability in upright standing before the perturbation (Fig. 3, top and middle panels).

### Effects of fatigue on joint kinematics

There were statistically significant effects of Condition and Time factors on *S*_Back_ (*χ*^2^_7_ = 16.2, *p* < 0.01). LocF condition induced a greater backward shoulder displacement after impact following fatigue exercise at Post 1 ($\overline{x}$: 4.0 cm ± 0.47 SEM) compared both to Pre ($\overline{x}$: 2.0 cm ± 0.55 SEM, *p* < 0.01) and to Post3 ($\overline{x}$: 2.2 cm ± 0.75 SEM, *p* < 0.05) (Fig. 3, bottom panel).

**Fig. 4:**
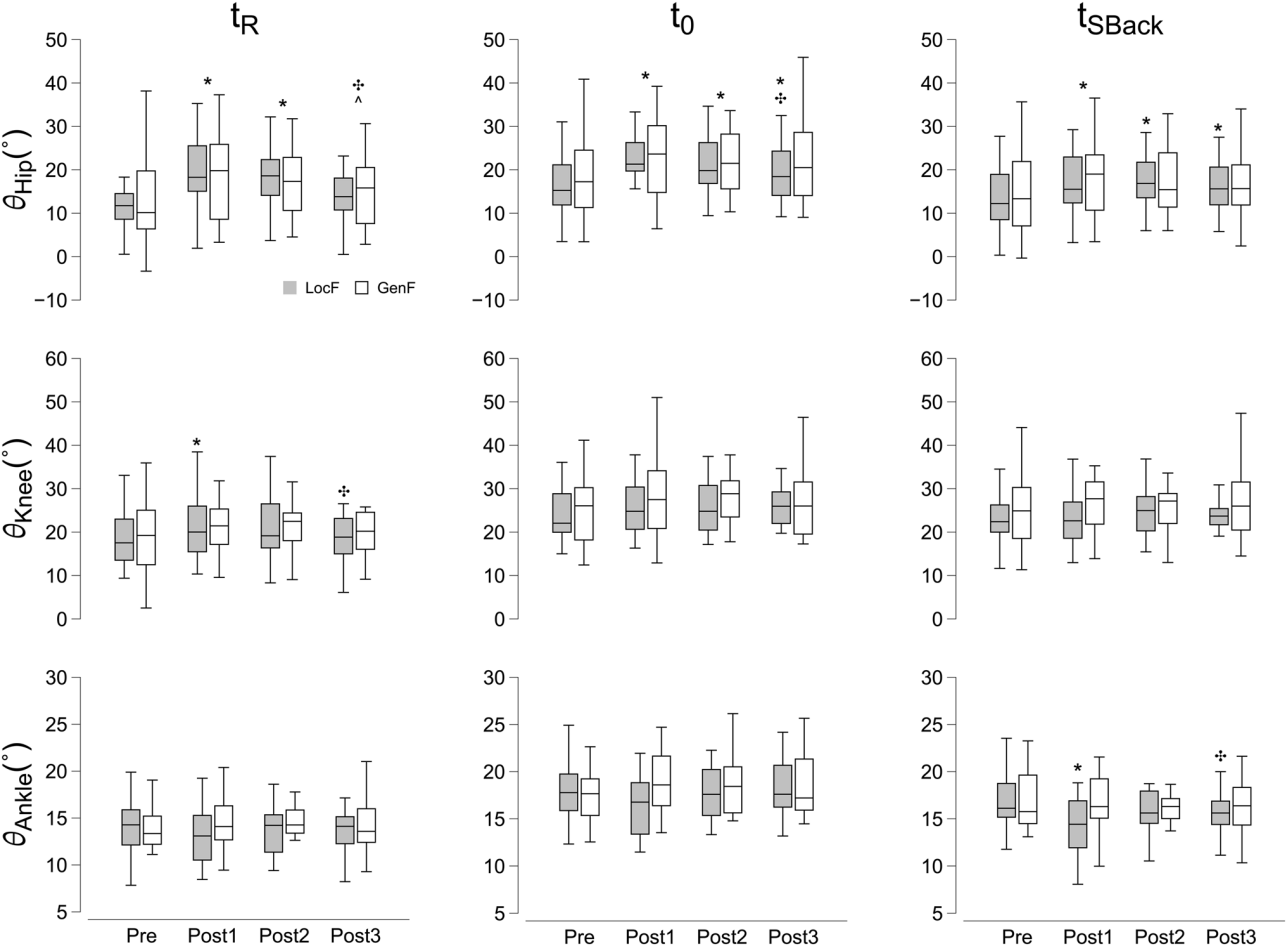
Box plots of results for kinematics of the hip (*θ*_Hip_), knee (*θ*_Knee_) and ankle (*θ*_Ankle_) joints at three different events during the trials: at the pendulum release (*t*_R_, left panels), at the pendulum impact (*t*_0_, panels in the center), and at the maximum shoulder displacement after impact (*t*_SBack_, panels on the right) before (Pre) and after (Post 1, Post2 and Post3) the fatiguing exercises (LocF and GenF). * Significant effects between Pre and Post1, Post2 or Post3; # significant effect between Post1 and Post2; ✣ significant effect between Post1 and Post3; ^ significant effect between Post2 and Post3. Significance level was set at *p* < 0.05

The two-way repeated measures ANOVA (Table 3) found significant effects of factor Time at the pendulum release (*t*_R_) for the hip joint angle (*θ*_Hip_), with post-hoc pairwise comparisons revealing a significant increase in hip flexion during Post1 and Post 2 compared to Pre and Post3 (*p* < 0.05). Similarly, the Friedman test’s pairwise comparisons showed that at release, knee joint angle (*θ*_Knee_) following LocF increased at Post1 compared to both Pre and Post3 (*p* < 0.05; Fig. 4, left panel). At the time point of impact (*t*_0_), the Friedman test found a significant increase in *θ*_Hip_ flexion angle across the first two post-exercise phases (Post1 and Post2; *p* < 0.05). Only for LocF, *θ*_Hip_ at Post3 was significantly higher than Pre and lower than Post1 (*p* < 0.01 and *p* < 0.05, respectively; Fig. 4, central panel). Regarding the joint angles at the time point of maximum shoulder displacement after impact (*t*_SBack_; Fig. 4, right panel), the Friedman test revealed a significant increase of *θ*_Hip_ for both LocF and GenF at Post1 compared to Pre (*p* < 0.001 and *p* < 0.01, respectively), while the difference from Pre during Post2 and Post3 was significant only for LocF (*p* < 0.001 and *p* < 0.01, respectively). The ANOVA revealed a significant effect of factor Time and Condition x Time for *θ*_Ankle_ at *t*_SBack_. Pairwise comparisons found an increased plantarflexion at Post1 following LocF, compared to both Pre and Post3 phases (*p* < 0.05).

**Table 3:** Two-way repeated measures ANOVA results for joint kinematics

Variable	Condition	Pre	Post1	Post2	Post3	Main effects			
							*F*—*χ*^2^	*p*	*η*^2^
*Release (t*_*R*_*)*									
*θ*_Hip_	LocF	12.43 ± 8.75	20.60 ± 10.51 *	19.42 ± 11.24 *	15.61 ± 11.05 **✣^**	Condition	*F*_1,13_ < 0.001	*p* = 0.986	–
	GenF	12.90 ± 11.03	20.43 ± 14.10 *	18.50 ± 12.14 *	16.36 ± 11.68 **✣^**	Time	*F*_3,39_ = 26.25	*p* < 0.001	*η*^2^ = 0.07
						Condition*Time	*F*_3,39_ = 0.58	*p* = 0.63	-
*θ*_Knee_	LocF	18.95 ± 6.66	21.40 ± 10.15 *	21.20 ± 7.16	20.0 ± 8.52	Friedman: Time (LocF only)	*χ*^2^_[7]_ = 13.56	*p* < 0.05	
	GenF	18.77 ± 8.65	22.69 ± 7.22 *	22.68 ± 8.32	20.95 ± 8.56				
*θ*_Ankle_	LocF	14.25 ± 2.98	13.49 ± 2.85	13.78 ± 2.85	13.92 ± 3.55 **✣**	Condition	*F*_1,13_ = 4.56	*p* = 0.318	–
	GenF	13.55 ± 3.48	14.48 ± 3.15	14.77 ± 3.03	14.26 ± 3.30	Time	*F*_3,39_ = 0.733	*p* = 0.843	–
						Condition*Time	*F*_3,39_ = 4.42	*p* = 0.104	–
*Impact (t*_*0*_*)*									
*θ*_Hip_	LocF	16.77 ± 7.99	23.73 ± 8.72 *	22.56 ± 9.86 *	20.54 ± 9.10 *✣	Friedman: Time (LocF & GenF)	*χ*^2^_[7]_ = 29.19	*p* < 0.001	
	GenF	18.25 ± 9.85	24.54 ± 13.16 *	23.18 ± 10.38 *	21.82 ± 10.46				
*θ*_Knee_	LocF	24.28 ± 6.36	25.47 ± 6.27	26.08 ± 5.99	26.55 ± 6.0	Condition	*F*_1,13_ = 1.39	*p* = 0.259	–
	GenF	25.54 ± 8.44	28.34 ± 9.58	28.54 ± 7.28	27.74 ± 8.08	Time	*F*_3,39_ = 2.23	*p* = 0.09	–
						Condition*Time	*F*_3,39_ = 0.843	*p* = 0.479	–
*θ*_Ankle_	LocF	18.05 ± 3.23	16.80 ± 3.48	18.76 ± 2.86	18.35 ± 3.01	Friedman: Time (LocF & GenF)	*χ*^2^_[7]_ = 11.79	*p* = 0.108	
	GenF	18.03 ± 3.60	18.65 ± 3.33	17.68 ± 3.40	18.44 ± 3.60				
*Max shoulder backward displacement (t*_*SBack*_*)*									
*θ*_Hip_	LocF	13.92 ± 8.09	18.08 ± 9.54 *	18.71 ± 10.06 *	17.42 ± 8.96 *	Friedman: Time (LocF & GenF)	*χ*^2^_[7]_ = 19.88	*p* < 0.01	
	GenF	14.58 ± 10.07	20.12 ± 12.33 *	18.52 ± 10.43	17.94 ± 10.61				
*θ*_Knee_	LocF	23.48 ± 6.49	23.16 ± 5.99	24.87 ± 5.66	24.63 ± 6.13	Condition	*F*_1,13_ = 1.84	*p* = 0.198	–
	GenF	24.89 ± 8.81	27.01 ± 9.0	26.45 ± 7.60	26.66 ± 8.29	Time	*F*_3,39_ = 1.97	*p* = 0.135	–
						Condition*Time	*F*_3,39_ = 1.84	*p* = 0.155	–
*θ*_Ankle_	LocF	16.59 ± 3.16	14.19 ± 3.27 *	15.72 ± 2.42	15.82 ± 2.57 **✣**	Condition	*F*_1,13_ = 2.01	*p* = 0.179	–
	GenF	16.81 ± 3.12	16.62 ± 3.13	16.31 ± 2.96	16.42 ± 3.15	Time	*F*_3,39_ = 8.45	*p* < 0.005	*η*^2^ = 0.03
						Condition*Time	*F*_3,39_ = 7.24	*p* < 0.005	*η*^2^ = 0.02

LocF: localized fatigue; GenF: general fatigue; *χ*^2^ Friedman chi-square; *η*^2^ eta squared effect size

◆Significant effects between Condition factor (GenF vs. LocF)

^*^Significant effects between Rest and Post1, Post2 or Post3

^**#**^Significant effect between Post1 and Post2

**✣**Significant effect between Post1 and Post3

**^**Significant effect between Post2 and Post3. Significance level was set at *p* < 0.05

### Effects of different fatiguing exercises on the anticipatory and compensatory postural EMG activity

**Fig. 5:**
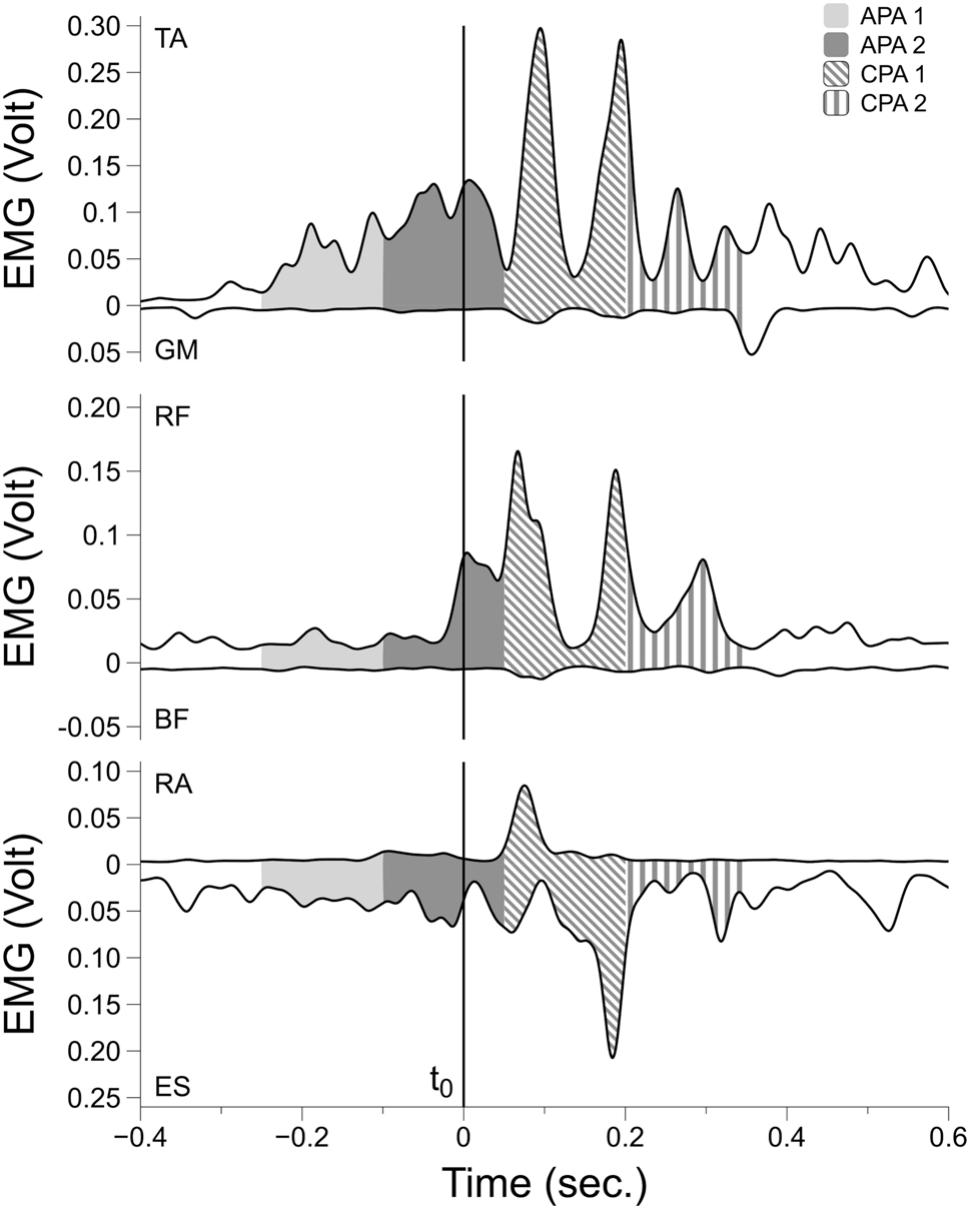
Filtered EMG traces of one trial in the Pre condition for a representative participant. The vertical solid line in the center of each panel corresponds to the impact time of the pendulum (*t*_0_). TA: tibialis anterior; GM: gastrocnemius medialis; RF: rectus femoris; BF: biceps femoris; RA: rectus abdominis; ES: erector spinae. Dorsal muscle activation patterns (GM, BF, ES) are shown inverted for ease of comparison. APA1, APA2, CPA1, CPA2 are highlighted in light gray, dark gray, oblique and vertical lines pattern respectively

Figure 5 shows EMG traces of one trial before the fatigue exercise (Pre) for a representative participant. Anticipatory and compensatory activity, appearing as bursts in the background EMG activity, was present in the ventral muscles (TA, RF, RA) and ES.

**Fig. 6:**
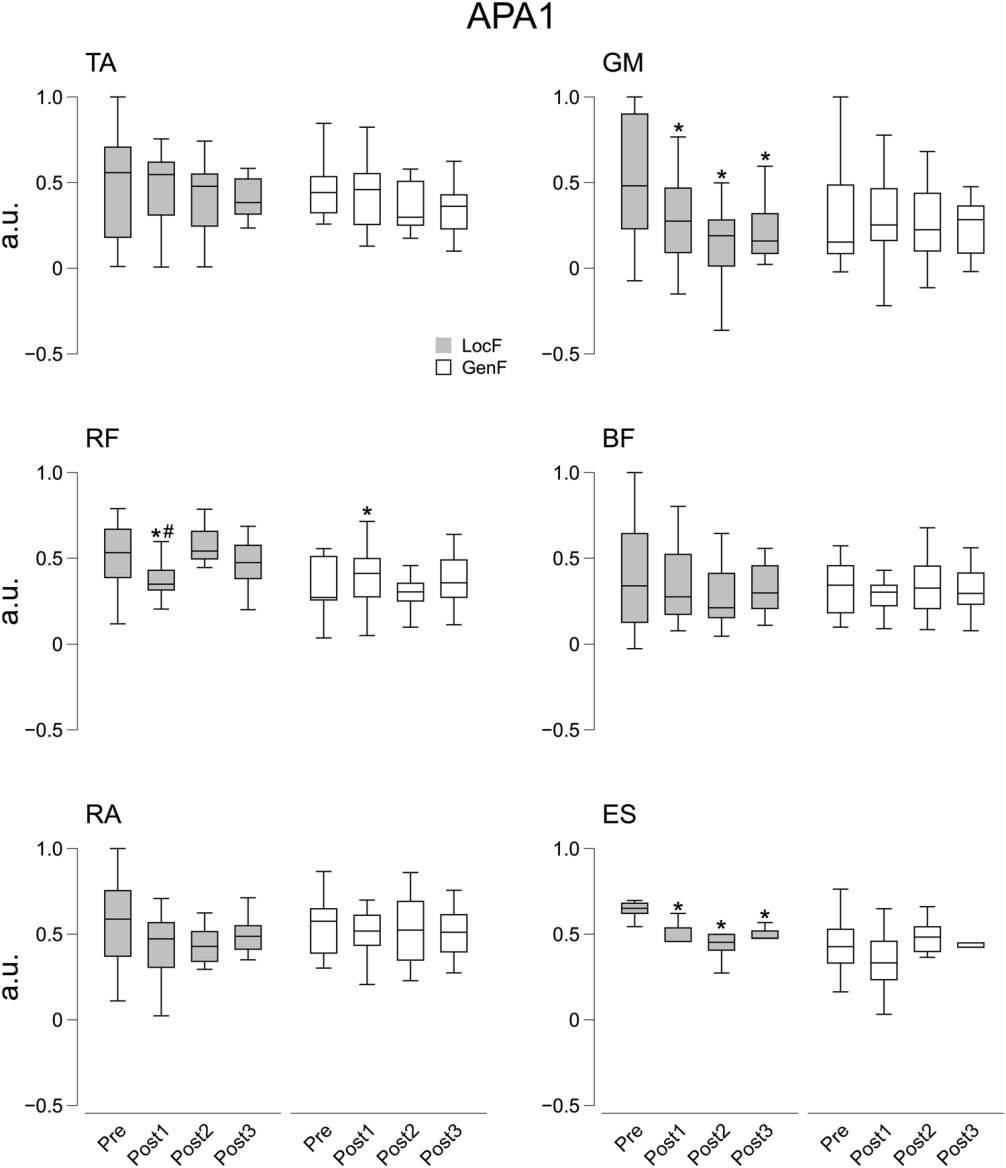
Integrals of EMG activity (∫APA1) during APA1 before (Pre) and after the fatigue exercise divided by the three Post time points (Post 1, Post2 and Post3). White and grey box indicate the GenF and LocF exercise protocol, respectively. TA: tibialis anterior; GM: gastrocnemius medialis; RF: rectus femoris; BF: biceps femoris; RA: rectus abdominis; ES: erector spinae. * Significant effects between Pre and Post1, Post2 or Post3; # significant effect between Post1 and Post2. Significance level was set at *p* < 0.05

**Fig. 7:**
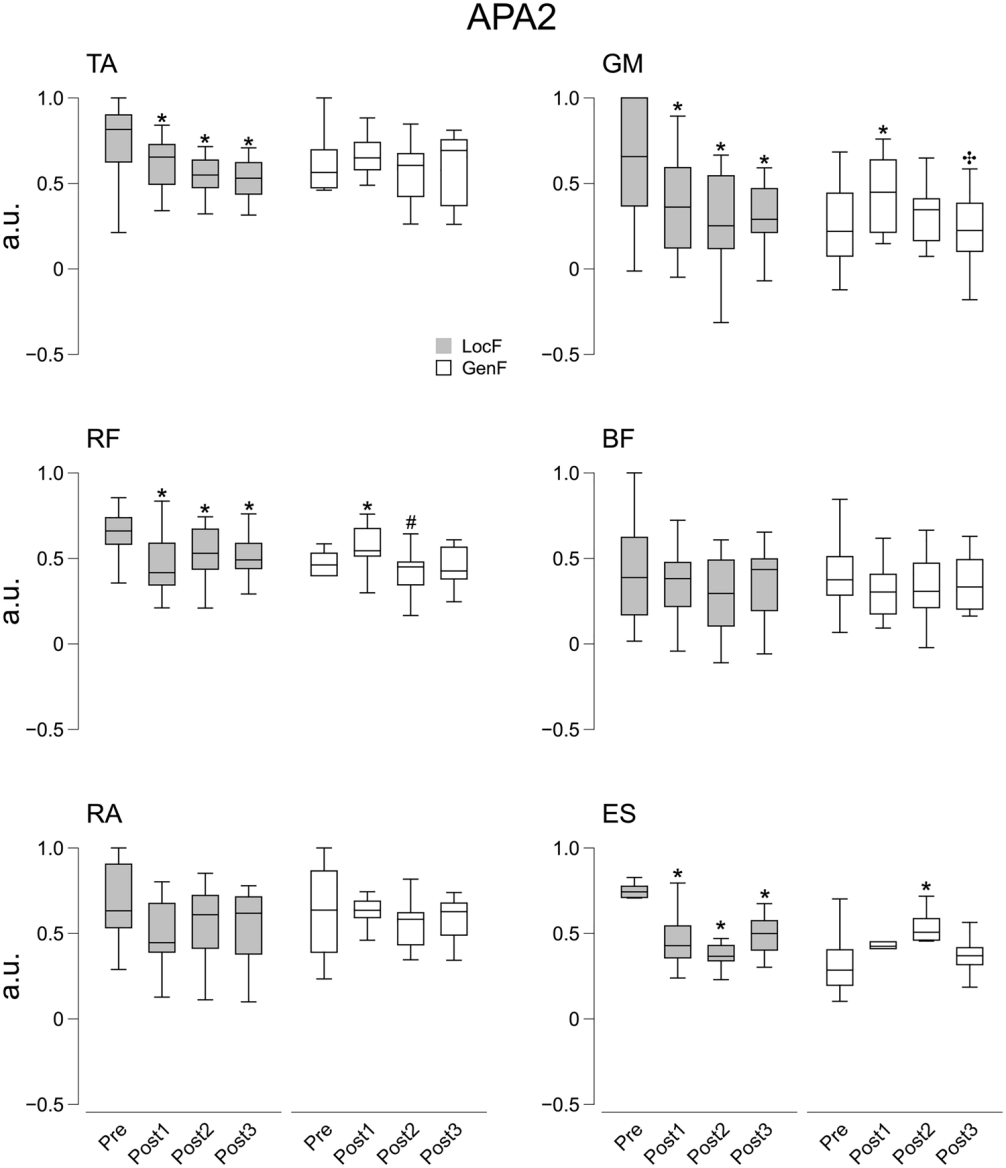
Integrals of EMG activity (∫APA2) during APA2 before (Pre) and after the fatigue exercise divided by the three Post time points (Post 1, Post2 and Post3). White and grey box indicate the GenF and LocF exercise protocol respectively. TA: tibialis anterior; GM: gastrocnemius medialis; RF: rectus femoris; BF: biceps femoris; RA: rectus abdominis; ES: erector spinae. * Significant effects between Pre and Post1, Post2 or Post3; # significant effect between Post1 and Post2. Significance level was set at *p* < 0.05

Statistical analysis found significant effects of the Condition and Time factor on GM during APA1 (*χ*^2^_7_ = 13.9, *p* < 0.05) and APA2 (*χ*^2^_7_ = 22.4, *p* < 0.01). Pairwise comparisons indicated a reduction in GM intensity at all the Post time points (Post1, Post2, and Post3) following LocF exercise during both APA1 (*p* < 0.05) and APA2 (*p* < 0.05). Conversely, GM showed higher activation at Post1 compared Pre and Post 3 after GenF exercise during APA2 (*p* < 0.01) (Figs. 6 and 7). There were statistically significant effects for Condition and Time (*χ*^2^_7_ = 19.0, *p* < 0.01) on TA during APA2. The anticipatory activity of TA during APA2 decreased after the LocF exercise in all three Post time points (*p* < 0.01), while not difference was observed with the GenF exercise condition. Significant effects were also observed for Condition and Time on RF and ES during APA1 (RF: *χ*^2^_7_ = 26.6, *p* < 0.001; ES: *χ*^2^_7_ = 24.9, *p* < 0.001) and APA2 (RF: *χ*^2^_7_ = 26.1, *p* < 0.001; ES: *χ*^2^_7_ = 23.6, *p* < 0.01). The post hoc pairwise comparisons showed a decreased ∫EMG of RF in all the Post time points during APA2 (*p* < 0.010) and in Post1 phase compared to Pre and Post2 during APA1 (*p* < 0.01) after performing the LocF protocol. By contrast, RF ∫EMG increased at Post1 compared to Pre during APA1 (*p* < 0.05), as well as compared to Post2 during APA2 following the GenF exercise (*p* < 0.05). ES muscle deactivation was observed only after the LocF exercise for the Post time points during both APA1 (*p* < 0.05) and APA2 (*p* < 0.05) (Figs. 6 and 7).

**Fig. 8:**
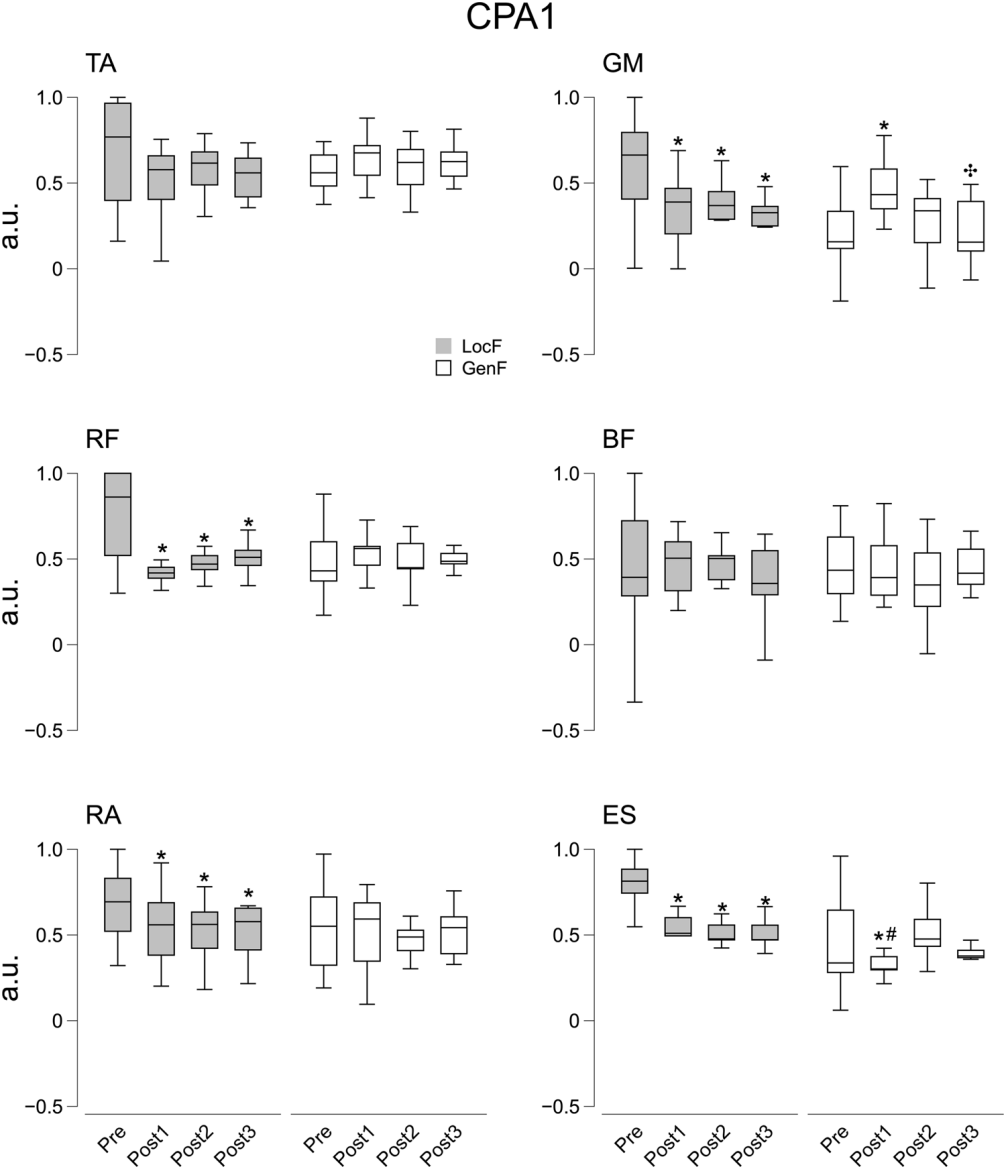
Integrals of EMG activity (∫CPA1) during CPA1 before (Pre) and after the fatigue exercise divided into the three Post time points (Post 1, Post2 and Post3). White and grey box indicate the GenF and LocF exercise protocol respectively. TA: tibialis anterior; GM: gastrocnemius medialis; RF: rectus femoris; BF: biceps femoris; RA: rectus abdominis; ES: erector spinae. * Significant effects between Pre and Post1, Post2 or Post3; # significant effect between Post1 and Post2. Significance level was set at *p* < 0.05

**Fig. 9:**
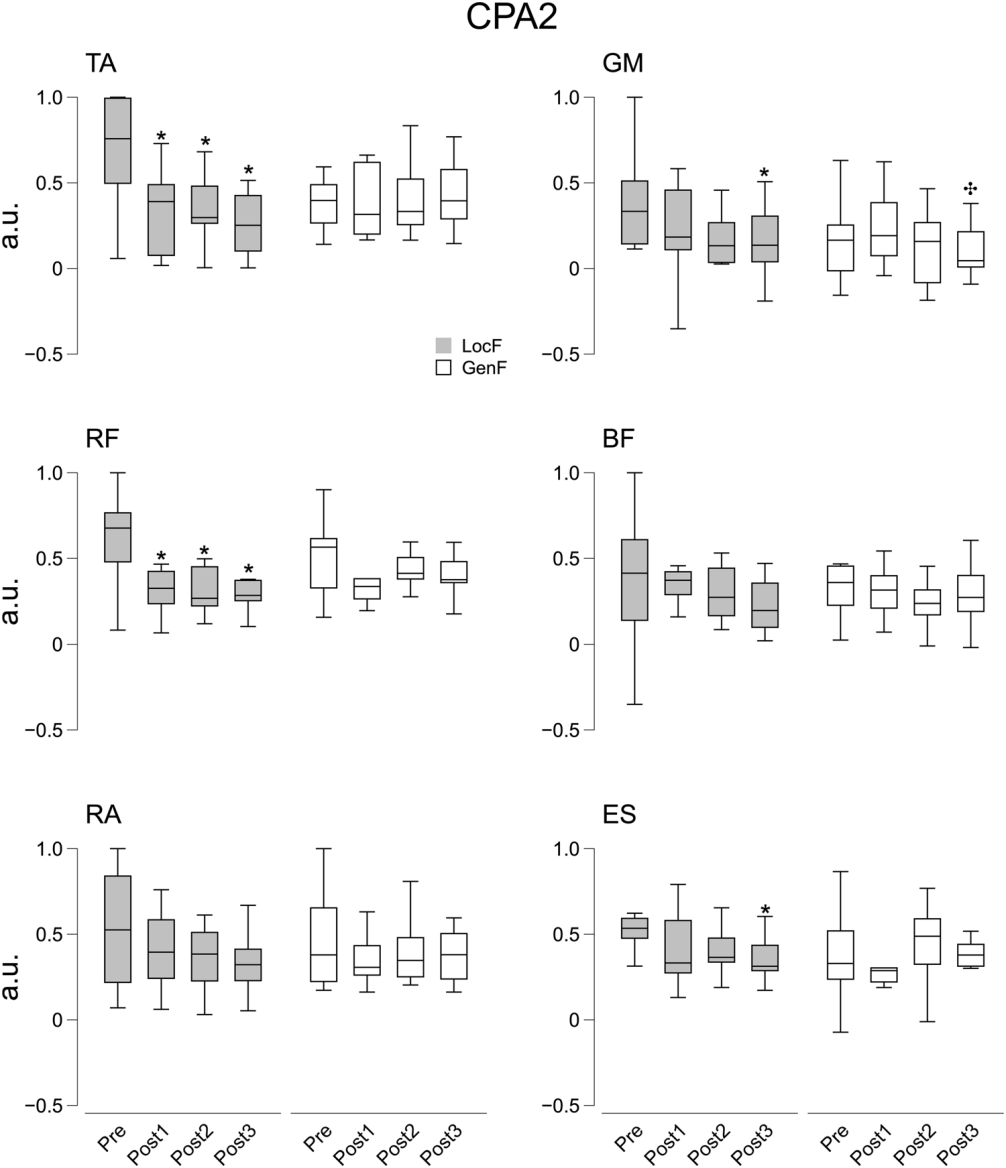
Integrals of EMG activity (∫CPA2) during CPA2 before (Pre) and after the fatigue exercise divided into the three Post time points (Post 1, Post2 and Post3). White and grey box indicate the GenF and LocF exercise protocol respectively. TA: tibialis anterior; GM: gastrocnemius medialis; RF: rectus femoris; BF: biceps femoris; RA: rectus abdominis; ES: erector spinae. * Significant effects between Pre and Post1, Post2 or Post3; # significant effect between Post1 and Post2. Significance level was set at *p* < 0.05

As far as the compensatory activity (∫CPA) is concerned, significant effects were found for Condition and Time factors on the ventral muscles RF (*χ*^2^_7_ = 22.6, *p* < 0.01) and RA (*χ*^2^_7_ = 15.9, *p* < 0.05) during CPA1, and on TA (*χ*^2^_7_ = 15.9, *p* < 0.05) and RF (Time: *F*_1.45,18.9_ = 17.6, *p* < 0.001, *η*^2^ = 0.39) during CPA2. There were statistically significant effects of Condition and Time factors on dorsal muscle GM and ES at both CPA1 (GM: *χ*^2^_7_ = 18.9, *p* < 0.01; ES: *χ*^2^_7_ = 34.0, *p* < 0.001) and CPA2 (GM: *χ*^2^_7_ = 15.4, *p* < 0.05; ES: *χ*^2^_7_ = 20.6, *p* < 0.01) epochs. The post hoc showed a systematic significant decrease of ∫CPA after LocF exercise in the three Post time phases for RF (*p* < 0.01), RA (*p* < 0.05), GM (*p* < 0.05), ES (*p* < 0.01) muscles during CPA1, and for TA (*p* < 0.05) and RF (*p* < 0.05) muscles during CPA2 epoch. In addition, decreased ∫CPA in GM and ES muscles was observed for Post3 compared to Pre during CPA2 (*p* < 0.05). GenF exercise resulted in a significant increase of ∫CPA in the GM muscle for Post 1 compared to Pre and Post3, and only to Post3 during CPA1 (*p* < 0.01) and CPA2 (*p* < 0.01) epochs, respectively. By contrast, there was a decrease of ∫CPA in the ES muscle for Post1 compared to Pre and Post2 during CPA1 (*p* < 0.05) following to GenF exercise (Figs. 8 and 9).

A significant effect was found on activation onset for the Muscle factor (*F*_2,26_ = 17.7, *p* < 0.001, *η*^2^ = 0.27). On average the TA muscle activated earlier (Onset_TA_: − 389 ms ± 20 SEM) than RF and RA muscle (Onset_RF_: − 242 ms ± 18 SEM, Onset_RA_: − 265 ms ± 21 SEM). The main factors Condition and Time did not show any significant effect on muscles’ onsets.

### Changes in co-activation indexes after fatiguing exercise

**Fig. 10:**
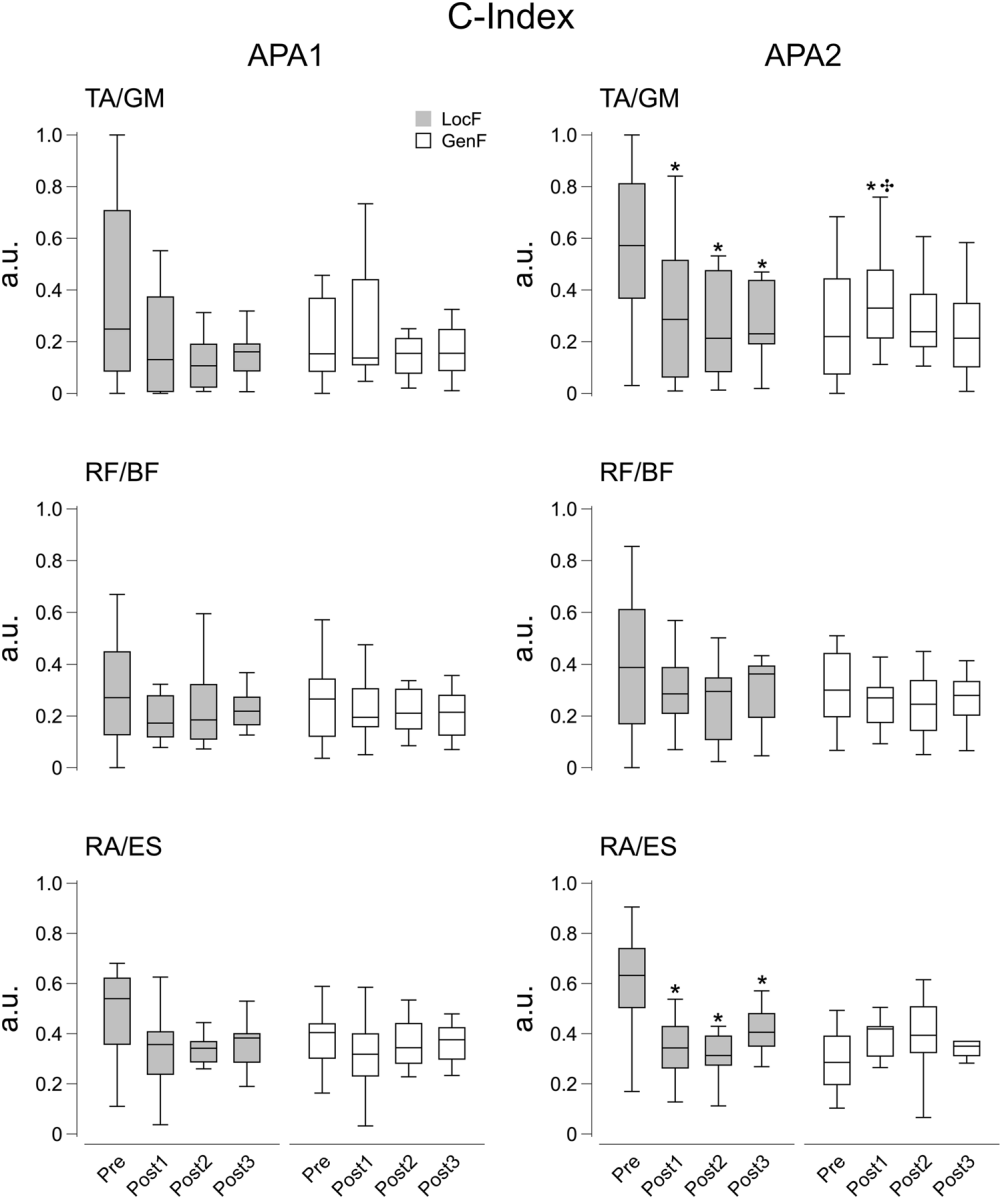
C-Index values for APA1 (left panels) and APA2 (right panels) for the agonist–antagonist pairs acting at the ankle (TA/GM), knee (RF/BF), and hip (RA/ES) joints before (Pre) and after the fatigue exercise divided into the three Post time points (Post 1, Post2 and Post3). White and grey box plots represent GenF and LocF exercise conditions respectively. * Significant effects between Pre and Post1, Post2 or Post3; ✣ significant effect between Post1 and Post3. Significance level was set at *p* < 0.05

The repeated measures analysis found significant effects for the Condition and Time factor on the C-Index (*χ*^2^_7_ = 28.6, *p* < 0.001) in the TA/GM pair during APA2. The pairwise comparisons showed a decrease in C-Index in the TA/GM muscle pair after LocF exercise over the three Post phases (*p* < 0.001), while following GenF exercise there was an increase in C-Index at Post1 compared to Pre and Post3 (*p* < 0.01) (Fig. 10). No significant effects were found on C-Index for RF/BF and RA/ES muscle pairs during APA2, as well as for all the muscle pairs during APA1.

**Fig. 11:**
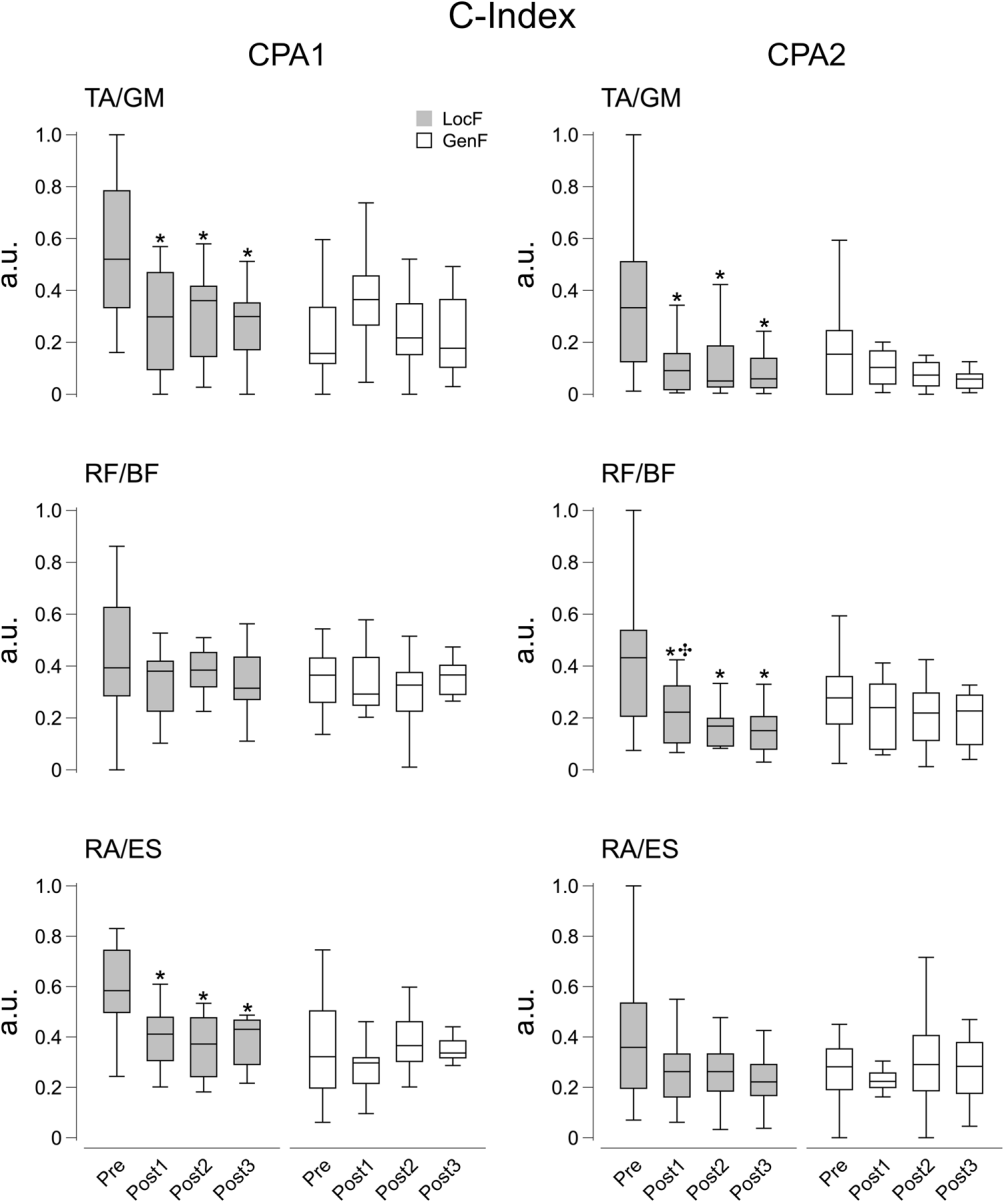
C-Index values for CPA1 (left panels) and CPA2 (right panels) for the agonist–antagonist pairs acting at the ankle (TA/GM), knee (RF/BF), and hip (RA/ES) joints before (Pre) and after the fatigue exercise divided into the three Post time points (Post 1, Post2 and Post3). White and grey box plots represent GenF and LocF exercise conditions respectively. * Significant effects between Pre and Post1, Post2 or Post3; ✣ significant effect between Post1 and Post3. Significance level was set at *p* < 0.05

With regards to the compensatory strategies, Condition and time factors showed significant effects on the C-Index of TA/GM pairs for CPA1 (*F*_1.8,23.4_ = 10.4, *p* < 0.001, *η*^2^ = 0.24) and CPA2 (*χ*^2^_7_ = 17.3, *p* < 0.05). During both the CPA1 and CPA2 epochs, TA/GM co-activation decreased over all the Post phases after the LocF exercise (*p* < 0.05). There was a significant effect of Condition and Time on C-Index for the RA/ES pair during CPA1 (*χ*^2^_7_ = 25.6, *p* < 0.001) showing a decrease of the co-activation after LocF for the Post phases (*p* < 0.01) (Fig. 11). The RF/BF muscle pair showed a significant effect of Condition and Time on the C-Index for CPA2 (*χ*^2^_7_ = 22.0, *p* < 0.01).

## Discussion

Our study revealed a distinct reorganization of postural adjustments following general and localized NMF. General fatigue, induced by high cardiometabolic effort, led to a short-lived increased EMG activity and co-activation in the lower limb muscles, suggesting strategic adjustments by the CNS to maintain stability. In contrast, localized NMF resulted in decreased muscle activation and co-activation across both fatigued and non-fatigued muscles, indicating a CNS-mediated adaptation likely due to altered proprioceptive feedback. Additionally, localized NMF caused persistent kinematic changes in the lower limb joints, while the effects of general fatigue were more transient, aligning with quicker recovery of cardiometabolic parameters. These findings underscore the CNS's role in adapting postural control strategies based on the type of fatigue experienced.

### Alteration of upright steady-state postural stability following the fatigue exercises

From a biomechanical viewpoint, the bipedal quiet standing posture requires individuals to control the sway of their CoP to keep the projection of their center of mass on the ground within the base of support (Winter et al. 1998; Peterka 2002, 2018). In this study, we found an increase in the variability (RMS_CoP_) and the mean velocity (Vel_CoP_) of the CoP sway in the two-dimensional directions immediately before the perturbation occurrence after both fatigue exercises, and these alterations persisted throughout all the recovery phases (Fig. 3). The findings indicate that fatiguing exercise impacts upright steady-state postural stability, as evidenced by increased variability and an accelerated center of pressure (CoP) sway. These changes are associated with postural compensations necessitated by sensorimotor deficits induced by fatigue (Paillard 2012). An additional potential cause of alterations in CoP-related measures is the observed increase in ventilation during both exercises (see Supplementary Fig. S2), which may have independently influenced postural stability (Paillard 2012). Previous studies have reported reduced performance during steady-state upright standing, following either localized or general NMF induced by exhaustive exercise. Specifically, increased displacement, RMS and velocity of the CoP trajectory were found in studies where fatigue was induced in ankle joint muscles (Yaggie and McGregor 2002; Gimmon et al. 2011; Barbieri et al. 2019; Degache et al. 2020). The decline in postural performance following NMF is likely related to the alteration of proprioceptive feedback (Boyas and Guével 2011; Wright and Arnold 2012), position sense (Proske 2019) and deterioration of muscle contractility (Ledin et al. 2004; Vuillerme et al. 2006). Similarly, a decrease in postural stability has been also reported with general fatigue that mobilizes or solicits a large part of body musculature such as during running (Nardone et al. 1997, 1998; Bove et al. 2007), cycling (Mello et al. 2010), or ergometer exercise (Lyu et al. 2021). The decline in postural stability subsequent to general fatiguing exercise is likely associated with deficiencies in central integration/processing (Wiest et al. 2011), altered metabolic homeostasis (Strang et al. 2008; Paillard 2012), and impaired sensitivity of sensory receptors (Nardone et al. 1997). These factors, particularly central integration/processing and metabolic homeostasis may have played a critical role in the increased postural sway observed following exhaustive exercise with the arm ergometer in our study.

### Changes of postural adjustments after fatiguing exercises

The postural adjustments exhibited distinct reorganization following either general (exhaustive exercise with high cardiometabolic effort) or localized NMF. Specifically, we observed a significant increase in EMG amplitude for the knee extensors (RF) and ankle plantar flexors (GM), along with augmented co-activation at the ankle joint muscles during the latter phase of APAs after exhaustive exercise. General fatigue was induced through a step-incremental test on an arm ergometer, limiting the involvement of postural muscles in the lower limb. Therefore, the adaptive modifications observed in the APAs are unlikely to be attributed to exercise-induced inability to activate these muscles properly. Instead, these adaptive muscular changes likely represent a strategic tuning in posture to proactively engage the neuromuscular system in counteracting the diminished stability during an upright stance due to fatigue (Paillard 2012; Zemková and Hamar 2014). This is in accordance with the interpretation of results from a previous study, where the anticipated onset of APA following strenuous aerobic exercise (Strang et al. 2008) was suggested to be a functional modulation of the CNS to maximize stability. In the present study, adaptive changes in postural muscles were facilitated by an additional kinematic modification at the hip joint, leading to increased flexion both before and upon impact, with this correction persisting beyond the occurrence of the impact. This adaptation aims to further prime the entire body for the onset of external perturbation, given the precarious nature of balance subsequent to exhaustive exercise.

We did not observe significant alterations during the CPA phase as a consequence of general fatiguing exercise. These findings contrast with those of a prior study by Lyu et al. (2021), where general fatigue induced with a rowing ergometer led to greater co-activation in the trunk and hamstring muscles during CPA subsequent to the initiation of bilateral arm lifting movements (Lyu et al. 2021). The differing results in the present study may be ascribed to specific characteristics of the experimental protocol, adopting an external perturbation that likely prompted the CNS to predominantly adapt anticipatory adjustments to counteract the significant mechanical impact of the pendulum, whereas Lyu et al. (2021) used self-initiated actions. Indeed, it has been demonstrated that the CNS employs distinct APAs and CPAs strategies to optimize postural stability when responding to perturbations of comparable magnitude applied to various body regions (Chen et al. 2017).

As far as the effects of localized NMF are concerned, a distinctly different pattern of changes was observed in APAs, and CPAs compared to the general fatigue. The primary difference observed was associated with a general decrease in EMG activity and co-activation, observed not only at the ankle joint muscles where NMF was induced but also at the proximal joint muscles. Specifically, it was found reduced activity in the TA muscle during late APAs and CPAs, as well as during both APAs phases and early CPAs in the GM muscle throughout all the recovery periods. These results are in line with a previous study that investigated the effects of localized NMF on balance recovery following a postural perturbation (Davidson et al. 2009), where the authors observed an increase in center of mass measures of displacement, and at the same time a reduction in CoP-based measures, suggesting a reduction in net torque used to control the angular momentum of the body. Additionally, a decrease in EMG activity was noted in non-fatigued postural muscles, including the RF during late APAs and CPAs, along with the ES and RA during both APAs and early CPAs phases, respectively (Figs. 6, 7 and 8). As a result, the decrease in EMG activity led to a reduction in the co-activation of muscles at the ankle joint during late APAs and compensatory CPAs, as well as at the hip and knee joints during late APAs and CPAs, respectively (Figs. 10 and 11). The decrease in muscular activity observed in non-fatigued muscles can be attributed to the concept of non-local muscle fatigue (Halperin et al. 2015). The neuromuscular fatiguing protocols can induce changes in the metabolic environment of the working muscles, resulting in the activation of group III and IV muscle afferents (Amann 2011, 2012; Amann et al. 2013). These muscle afferents, through a feedback loop, exert an inhibitory effect on the central nervous system, leading to reductions in the central drive to the working muscles and potentially affecting non-exercised muscles as well (Amann 2011, 2012; Amann et al. 2013; Sidhu et al. 2014; Laginestra et al. 2021). To support the notion of an inhibitory effect on the central drive, Sidhu et al. (2014) investigated how cycling to exhaustion with and without lower limb sensory feedback affected force production and elbow flexor activation. They found reduced responsiveness of motor cortical cells and spinal motoneurons during normal conditions, contrasting with no change when lower limb muscle afferents were blocked (Sidhu et al. 2014). This suggests that group III and IV muscle afferents inhibit corticospinal motor pathways in the upper limb during lower body muscle fatigue. While it has been suggested that the non-local effects of group III and IV afferents are unlikely (Kennedy et al. 2014) and ongoing debate exists regarding its general effects (Behm et al. 2021), substantial research has demonstrated the effects of non-local muscle fatigue on postural control. Specifically, following localized neuromuscular fatigue, alterations in activation patterns of non-fatigued postural muscles have been reported in previous studies (Strang et al. 2009; Singh and Latash 2011; Lyu et al. 2021; Nardon et al. 2024b), proving support for a centrally mediated adaptation of the CNS aimed at preserving postural stability (Vuillerme et al. 2002; Morris and Allison 2006; Strang and Berg 2007; Kanekar et al. 2008; Strang et al. 2009; Mezaour et al. 2010; Nardon et al. 2024b).

To our knowledge, only two studies (Strang et al. 2008; Lyu et al. 2021) have investigated the effects of general fatigue on anticipatory postural adjustments (APAs) and compensatory postural adjustments (CPAs). Strang et al. (2008) observed that exhaustive running exercise led to earlier APAs in the paraspinal muscles during rapid bilateral arm raising, with no timing effect noted in other postural muscles (Strang et al. 2008). Similarly, when inducing general fatigue with a rowing ergometer, Lyu et al. (2021) found no significant alterations in the onset and EMG amplitude during the APA phase preceding the initiation of bilateral arm lifting movements. However, a significant increase in co-activation at the trunk and hamstring muscles was noted during the CPA phase following the fatiguing exercise (Lyu et al. 2021). Our results confirmed a lack of alteration in the timing of APAs following general muscular exercise. However, it was found a significant increase in EMG amplitude for the knee extensors (RF) and ankle plantar flexors (GM), as well as augmented co-activation at the ankle joint muscles during the late phase of APA. In contract with Lyu et al. (2021), no significant changes occurred during the compensatory phase.

### Fatigue-induced changes in muscle co-activation profiles

In addition to individual muscle activation, the approach of computing muscle co-activation at joint level has been widely used (Singh and Latash 2011; Bertucco et al. 2021; Cesari et al. 2022; Pascucci et al. 2023; Nardon et al. 2024b). Interpretation of muscle co-activation during upright standing has some contradictory aspects. Traditionally, the simultaneous activation of agonist and antagonist muscles acting on the same joint has been recognized to enhance postural stability by increasing joint stiffness (Nielsen and Kagamihara 1992; Lee et al. 2006). This view has been recently criticized because feet are not typically anchored to the ground during standing, meaning that biomechanically none of the ends of the chain is fixed in space (Latash 2018). In fact, few recent studies reported impaired stability when the co-activation level was purposefully increased (Yamagata et al. 2019, 2021). Moreover, a recent study reported a decrease in the co-activation of muscle pairs following the experimental manipulation of postural threat (Cesari et al. 2022). When participants experienced an increased risk of falling, muscle co-activation during action preparation decreased, suggesting that the co-contraction itself could induce a potential internal perturbation which puts balance under risk (Chen et al. 2017; Yamagata et al. 2019, 2021).

The reduction observed in muscle co-activation levels following LocF might result from fatigue-induced alteration in proprioceptive feedback (Vuillerme and Boisgontier 2008), changing how participants relied on visual information and inducing a re-calibration of neural drive to the muscle (Takahashi et al. 2006; Paillard 2012). Furthermore, as recently reported by Cesari et al., participants tend to reduce muscle co-activation in preparation to an external perturbation to limit joint stiffness and its potential negative effect on standing balance (Cesari et al. 2022). The differing results in terms of muscle co-activation strategies between the two fatiguing protocols and the fact that such alterations extend to non-fatigued muscles suggest that fatigue-induced adaptation takes place at the level of the CNS. Our results closely align with those of previous studies examining the effects of localized fatiguing protocols on postural control (Singh and Latash 2011; Lyu et al. 2021; Nardon et al. 2024b).

### Adaptations in joint kinematics induced by fatigue and the subsequent recovery processes

Localized neuromuscular fatigue caused a larger post-perturbation backward displacement of the shoulder during the early recovery phase (Post1), underlining the acute exercise-induced disturbance in postural control. Conversely, no effect of general fatigue was seen on shoulder displacement. The lack of effect could be potentially linked to the pronounced perception of effort—and thus of potential risk of falling awareness—following the GenF protocol, resulting in a more conservative approach to stabilize posture. Using a similar perturbation protocol, Cesari et al. reported a decrease in post-perturbation backward displacement of the shoulder when postural threat was increased (Cesari et al. 2022). Interestingly, both fatiguing protocols induced changes in knee and hip joint angles across the recovery phases. Furthermore, the localized NMF also led to a pronounced co-variation in the lower limb joints (Fig. 4), which persisted throughout the recovery phases. This enduring effect of non-local muscle fatigue in the associated joint muscles was evidenced by the APA and CPA activities, along with co-activation levels, which remained significantly different from the pre-fatigue trials. This further demonstrates that afferent information from the persistent peripheral changes prompted the central nervous system to continue modulating the postural response, regardless of which postural muscle was fatigued (Kanekar et al. 2008; Halperin et al. 2015; Nardon et al. 2024b). Indeed, previous research has documented similar adverse effects on muscle activity and co-activation related to postural control strategies following neuromuscular fatigue in the plantar flexor muscles, with these effects persisting for more than 10 min (Strang et al. 2008; Kennedy et al. 2011, 2012). In contrast, such sustained variation of kinematic and muscular strategies was observed only during the early recovery phase following generalized fatigue exercising. Our findings align with previous studies that reported adverse effects on postural stability following intensive cardio-metabolic exercise, yet these effects were relatively short-lived, with significant improvement observed within 10 min of recovery compared to the non-fatigue condition (Fox et al. 2008; Steinberg et al. 2016).

Fatigue-induced alterations in joint kinematics have been previously reported following both general (Derrick et al. 2002; Nardon et al. 2024a) and local (Christina et al. 2001; Kellis and Liassou 2009) fatiguing exercise. Previous studies, however, reported alterations in joint angles during dynamic movements, making it hard to infer whether these changes were due to alteration in afferent feedback, impaired sensorimotor integration or suboptimal efferent drive from the CNS to the muscles.

To our knowledge, our study is the first that monitored the post-exercise recovery of cardio-metabolic parameters, simultaneously assessing the adaptation of postural control strategies following two distinct fatiguing protocols. Despite the known influences of fatigue-induced neurophysiological alterations on motor performance (Paillard 2012; Carroll et al. 2017), it seems that the CNS implements adaptive strategies—adjusting motor command and motor planning to the novel, fatigued state (Takahashi et al. 2006)—which are independent from physiological recovery processes.

## Conclusions

To summarize, our results show that postural adjustments undergo distinct reorganization following both general and localized NMF. General fatigue, induced by exhaustive exercise, led to increased muscle activation and co-activation at the ankle joint during APAs, reflecting a strategic neuromuscular adaptation to counteract reduced stability. Conversely, localized NMF resulted in decreased muscle activity and co-activation, not only in fatigued muscles but also in non-fatigued ones, suggesting a broader central nervous system adaptation. Long-lasting post-exercise increases in postural instability have been observed following both fatiguing protocols. Similarly, adaptations in joint kinematics have been documented, with more persistent alterations following localized NMF. The differing impacts of general and localized fatigue on postural control highlight the CNS's role in modulating motor strategies based on the type of fatigue, independent of physiological recovery processes. Taken together, the results demonstrate that the CNS is able to cope with the perturbation of physiological homeostasis induced by NMF.

## Supplementary Information

Below is the link to the electronic supplementary material.

Supplementary file 1 (DOCX 218 KB)

Supplementary file 2 (DOCX 268 KB)

## Data Availability

The datasets generated during and/or analyzed during the current study are available from the corresponding author on reasonable request.

## Abbreviations

ANOVA: Analysis of variance
APA: Anticipatory postural adjustments
BF: Biceps femoris
C-index: Co-activation index
CNS: Central nervous system
CoP: Center of pressure
CPA: Compensatory postural adjustments
EMG: Electromyographic
ES: Erector spinae
GenF: General neuromuscular fatigue protocol
GM: Gastrocnemius medialis
HR: Heart rate
LocF: Localized neuromuscular fatigue protocol
MVC: Maximal voluntary contraction
NMF: Neuromuscular fatigue
Post: Post-exercise
Pre: Pre-exercise
RA: Rectus abdominis
RF: Rectus femoris
RMS_CoP_: CoP root mean square
RR: Respiratory rate
SEM: Standard error of the mean
*S*_Back_: Maximum shoulder displacement in the sagittal plane
TA: Tibialis anterior
*t*_0_: Timepoint of pendulum impact
*t*_R_: Timepoint of the pendulum release
*t*_SBack_: Timepoint of *S*_Back_
${\dot{\text{V}}\text{CO}}_{2}$: Carbon dioxide production
$$\dot{V}_{{\text{E}}}$$: Pulmonary ventilation
Vel_CoP_: CoP mean velocity
$${\dot{\text{V}}\text{O}}_{2}$$: Oxygen consumption
*θ*_Ankle_: Ankle joint angle in the sagittal plane
*θ*_Hip_: Hip joint angle in the sagittal plane
*θ*_Knee_: Knee joint angle in the sagittal plane
