# Supplementary material for: Effects of localized and general fatigue on postural adjustments coupling during predictable external perturbations
Source: Eur J Appl Physiol. 2025 Apr 5;125(9):2539–61. doi: 10.1007/s00421-025-05760-y (PMC12423267; doi:10.1007/s00421-025-05760-y)
Supplement: Supplementary file 1 — (DOCX 218 KB) [file 421_2025_5760_MOESM1_ESM.docx]

**Figure S1: Metabolic responses during fatiguing protocols**


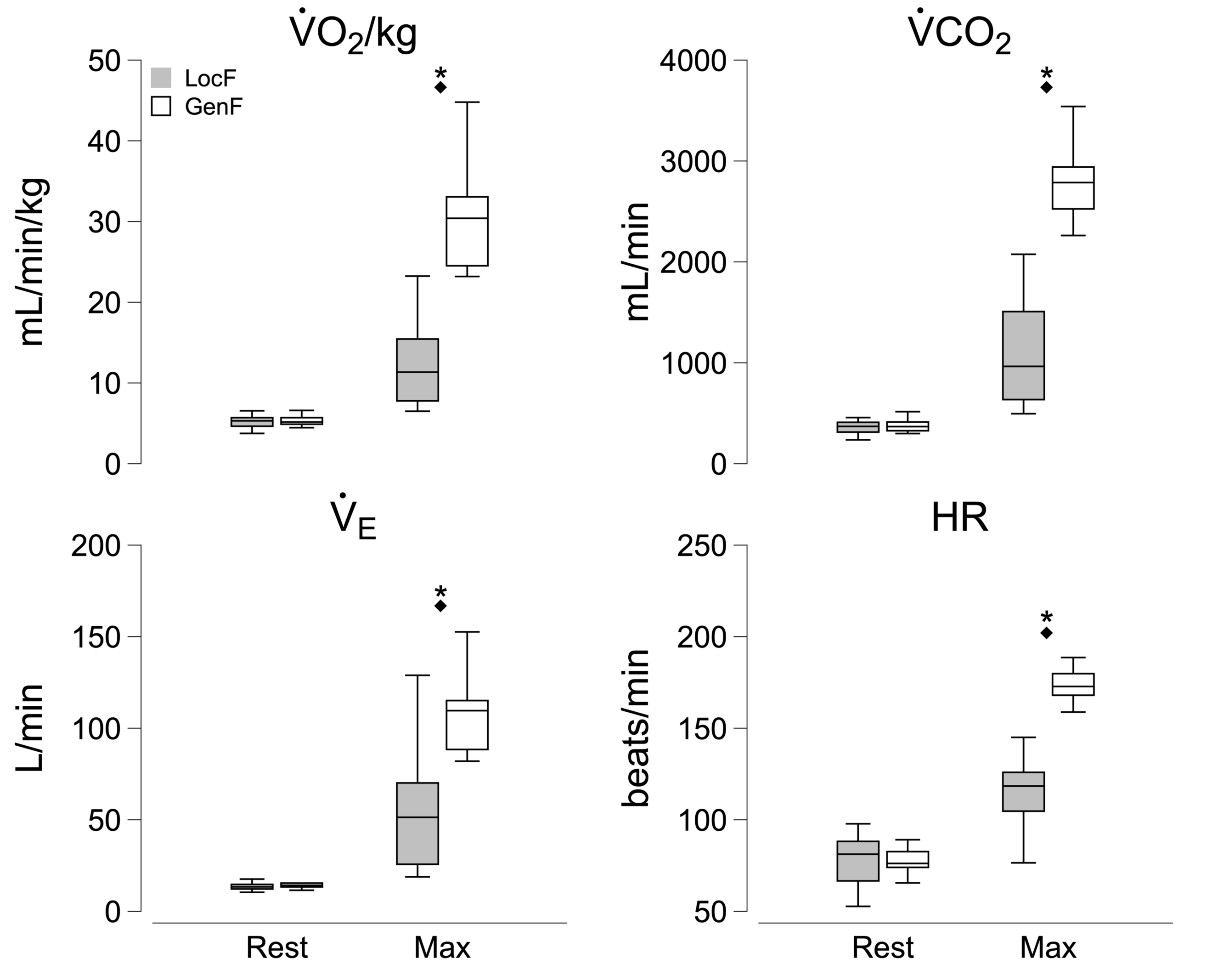


**S1.** Metabolic responses ($\dot{V}O$_2_/kg, $\dot{V}CO$_2_, $\dot{V}_{E}$, HR) at Rest and to exhaustion (Max) between GenF (white box) and LocF (grey box) fatigue exercises. ◆ Significant effects within Condition factor (GenF vs LocF); * significant effects within Time factor (Rest vs Max). Signiﬁcance level set at p < 0.05.
