# Supplementary material for: Effects of localized and general fatigue on postural adjustments coupling during predictable external perturbations
Source: Eur J Appl Physiol. 2025 Apr 5;125(9):2539–61. doi: 10.1007/s00421-025-05760-y (PMC12423267; doi:10.1007/s00421-025-05760-y)
Supplement: Supplementary file 2 — (DOCX 268 KB) [file 421_2025_5760_MOESM2_ESM.docx]

**Figure S2: Cardiopulmonary responses** **during the fatiguing protocols**


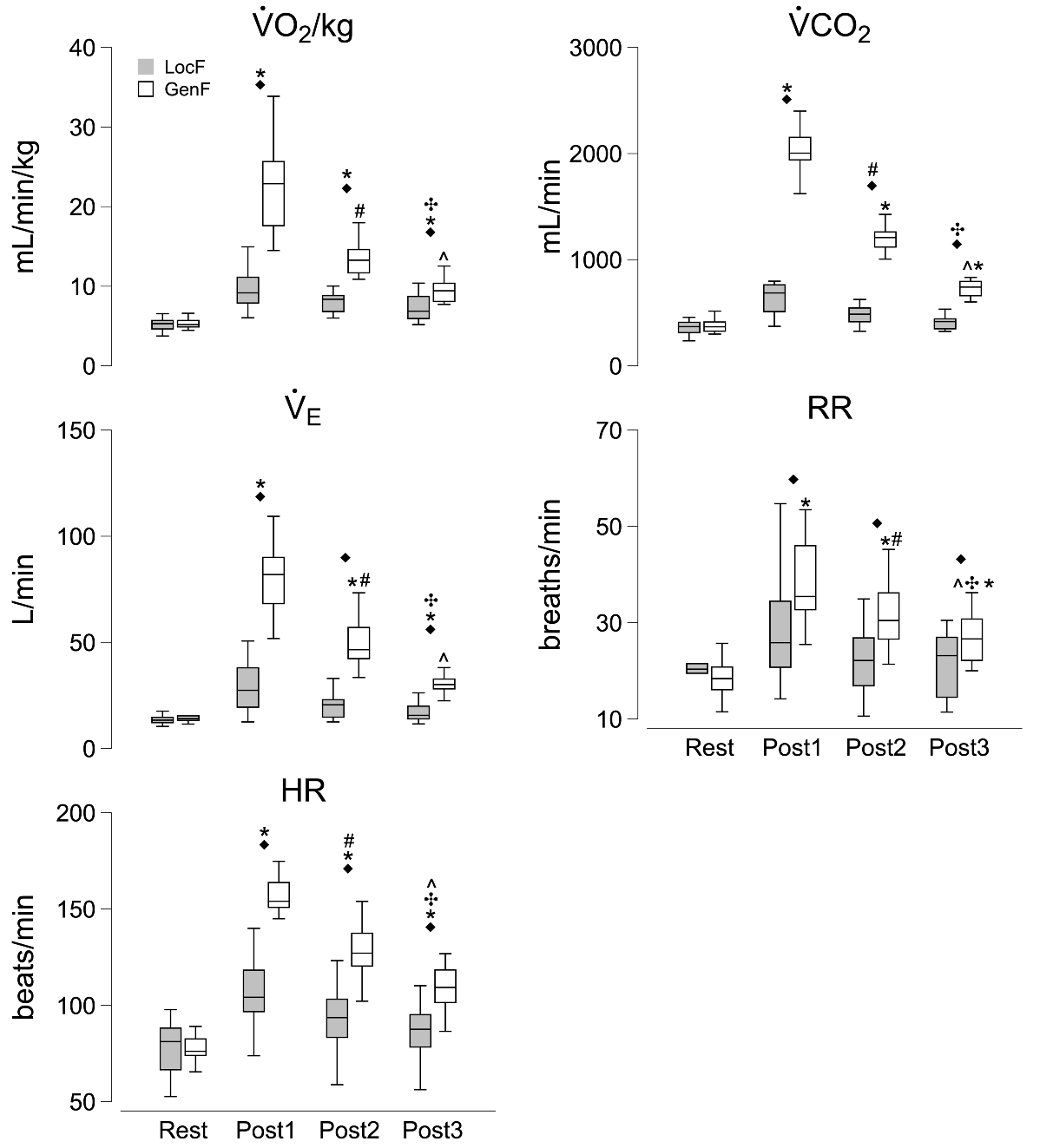


**S2.** Cardiopulmonary responses ($\dot{V}O$_2_/kg, $\dot{V}CO$_2_, $\dot{V}_{E}$, RR, HR) between GenF (white box) and LocF (grey box) fatigue exercises at Rest and at the Post phases (Post1, Post2 and Post3) while participants received the pendulum perturbation. ◆ Significant effects between Condition factor (GenF vs LocF); * significant effects between Rest and Post1, Post2 or Post3; # significant effect between Post1 and Post2; ✣ significant effect between Post1 and Post3; ^ significant effect between Post2 and Post3. Signiﬁcance level was set at p < 0.05.
